# The Interfield Strength Agreement of Left Ventricular Strain Measurements at 1.5 T and 3 T Using Cardiac MRI Feature Tracking

**DOI:** 10.1002/jmri.28328

**Published:** 2022-06-29

**Authors:** Sarah L. Ayton, Aseel Alfuhied, Gaurav S. Gulsin, Kelly S. Parke, Joanne V. Wormleighton, J. Ranjit Arnold, Alastair J. Moss, Anvesha Singh, Hui Xue, Peter Kellman, Matthew P. M. Graham‐Brown, Gerry P. McCann

**Affiliations:** ^1^ Department of Cardiovascular Sciences University of Leicester and the NIHR Leicester Biomedical Research Centre, Glenfield Hospital Leicester UK; ^2^ National Heart, Lung and Blood Institute, National Institutes of Health Bethesda Maryland USA

**Keywords:** cardiac MRI, field strength, left ventricular strain, myocardial deformation analysis

## Abstract

**Background:**

Left ventricular (LV) strain measurements can be derived using cardiac MRI from routinely acquired balanced steady‐state free precession (bSSFP) cine images.

**Purpose:**

To compare the interfield strength agreement of global systolic strain, peak strain rates and artificial intelligence (AI) landmark‐based global longitudinal shortening at 1.5 T and 3 T.

**Study Type:**

Prospective.

**Subjects:**

A total of 22 healthy individuals (mean age 36 ± 12 years; 45% male) completed two cardiac MRI scans at 1.5 T and 3 T in a randomized order within 30 minutes.

**Field Strength/Sequence:**

bSSFP cine images at 1.5 T and 3 T.

**Assessment:**

Two software packages, Tissue Tracking (cvi42, Circle Cardiovascular Imaging) and QStrain (Medis Suite, Medis Medical Imaging Systems), were used to derive LV global systolic strain in the longitudinal, circumferential and radial directions and peak (systolic, early diastolic, and late diastolic) strain rates. Global longitudinal shortening and mitral annular plane systolic excursion (MAPSE) were measured using an AI deep neural network model.

**Statistical Tests:**

Comparisons between field strengths were performed using Wilcoxon signed‐rank test (*P* value < 0.05 considered statistically significant). Agreement was determined using intraclass correlation coefficients (ICCs) and Bland–Altman plots.

**Results:**

Minimal bias was seen in all strain and strain rate measurements between field strengths. Using Tissue Tracking, strain and strain rate values derived from long‐axis images showed poor to fair agreement (ICC range 0.39–0.71), whereas global longitudinal shortening and MAPSE showed good agreement (ICC = 0.81 and 0.80, respectively). Measures derived from short‐axis images showed good to excellent agreement (ICC range 0.78–0.91). Similar results for the agreement of strain and strain rate measurements were observed with QStrain.

**Conclusion:**

The interfield strength agreement of short‐axis derived LV strain and strain rate measurements at 1.5 T and 3 T was better than those derived from long‐axis images; however, the agreement of global longitudinal shortening and MAPSE was good.

**Evidence Level:**

2

**Technical Efficacy:**

Stage 2

## Background

Myocardial deformation analysis provides global and regional information on left ventricular (LV) contraction and relaxation, enabling assessment of myocardial function in different planes. Deformation of myocardial segments (termed “strain”) is described in longitudinal, circumferential, and radial directions and represents a more integrated view of myocardial mechanics compared to LV ejection fraction (EF).^1^, ^2^, ^3^ Numerous studies have demonstrated the incremental diagnostic and prognostic value of myocardial strain assessment in different disease states, including heart failure, valvular heart disease, and following myocardial infarction.^4^, ^5^, ^6^, ^7^

Strain and strain rate measurements by speckle tracking echocardiography have been advocated for diagnosis of heart failure in the European Society of Cardiology guidelines^8^ and, given the increasing availability and accuracy of cardiac magnetic resonance imaging (MRI) studies, consideration must be given to the standardization of cardiac MRI‐derived strain indices. Currently, global longitudinal strain (GLS) is the most used measure clinically at present, however, in research both systolic and diastolic strain rate measurements are used as markers of LV function. Software developments now permit measurement of cardiac MRI‐derived LV strain from routinely acquired balanced steady‐state free precession (bSSFP) cine images. This has the potential to shorten scan times and streamline analyses compared to previously used methods, such as myocardial tissue tagging and phase velocity mapping.^9^ Strain derived from bSSFP methods has good agreement with speckle tracking echocardiography,^10^, ^11^ but agreement with cardiac MRI tagging methods has varied between studies, with radial strain showing the poorest agreement.^12^, ^13^, ^14^, ^15^, ^16^ Excellent test–retest reproducibility has been demonstrated at both 1.5 T and 3 T in different disease populations with two commercially available software packages used to derive strain from cardiac MRI bSSFP images.^17^

Further to measurement of strain, global longitudinal shortening (similar to long‐axis strain) can be measured from long‐axis bSSFP images using a recently developed artificial intelligence (AI) method. Global longitudinal shortening is derived by calculating the length of the LV from the apex to the midpoint of the mitral valve insertion points at diastole and systole.^18^ The AI approach involves fully automated detection and tracking of required landmarks and has shown promise in providing an efficient method to derive this measurement.^18^ Global longitudinal shortening has been proposed to be a simpler and faster approach to determine longitudinal function compared to longitudinal strain^19^, ^20^ and may overcome differences seen in strain measurements between postprocessing software vendors.^21^

The majority of studies reporting normal values for strain measurements from bSSFP cine images have used 1.5 T systems,^22^ but several have combined strain measures from 1.5 T and 3 T systems.^10^, ^12^, ^23^, ^24^ Interstudy repeatability of LV circumferential strain has previously been found to be better at 1.5 T than 3 T, which may be due to images at 3 T being more susceptible to artifact.^25^ However, the agreement of strain measurements, as well as global longitudinal shortening, at different field strengths has yet to be established. In a previous study, mean systolic strain measurements were higher at 1.5 T vs. 3 T and the interfield strength agreement was fair for global circumferential strain (GCS), but there was no agreement for global longitudinal strain (GLS).^26^ However, in this analysis, acquisitions at 1.5 T were performed sequentially after imaging at 3 T and short‐axis cine images at 1.5 T were obtained following adenosine stress and gadolinium contrast. Clinical and research MRI centers frequently utilize both 1.5 T and 3 T MRI systems, and therefore, patients and research participants may be scanned on either or both scanners during their investigation and treatment. Comparisons between the two field strengths are required to ensure measures between patients, and more importantly, when the same patient is scanned sequentially, are comparable. In addition, normal ranges may be needed for both field strengths. Assessment of strain and strain rate measures between field strengths is therefore required in a prospective and randomized manner.

The aims of this study were to assess the agreement of global systolic strain, peak strain rate measurements and AI landmark‐based global longitudinal shortening derived from cardiac MRI bSSFP cine images acquired at 1.5 T and 3 T. We hypothesized that global strain and shortening measured at 1.5 T and 3 T would have good agreement when assessed in the same subjects scanned sequentially in a randomized order.

## Methods

### Study Population

The study was approved by the United Kingdom National Research Ethics Service (19/YH/0392) and conducted according to the Declaration of Helsinki. All participants provided written informed consent. Twenty‐two healthy individuals were prospectively recruited to complete two cardiac MRI scans on the same day at 1.5 T and 3 T systems. The inclusion criteria were male or female individuals, aged 18 years or above, willing and able to give informed consent for participation in the study with sufficient understanding of written and verbal English to be able to consent and participate in study. Exclusion criteria were history of prior cardiovascular, respiratory, metabolic (including diabetes) or renal disease, any contra‐indication to MRI, including the presence of an implanted metal device or suspected metal foreign bodies, and pregnancy.

### Participant Flow and Randomization

**FIGURE 1:**
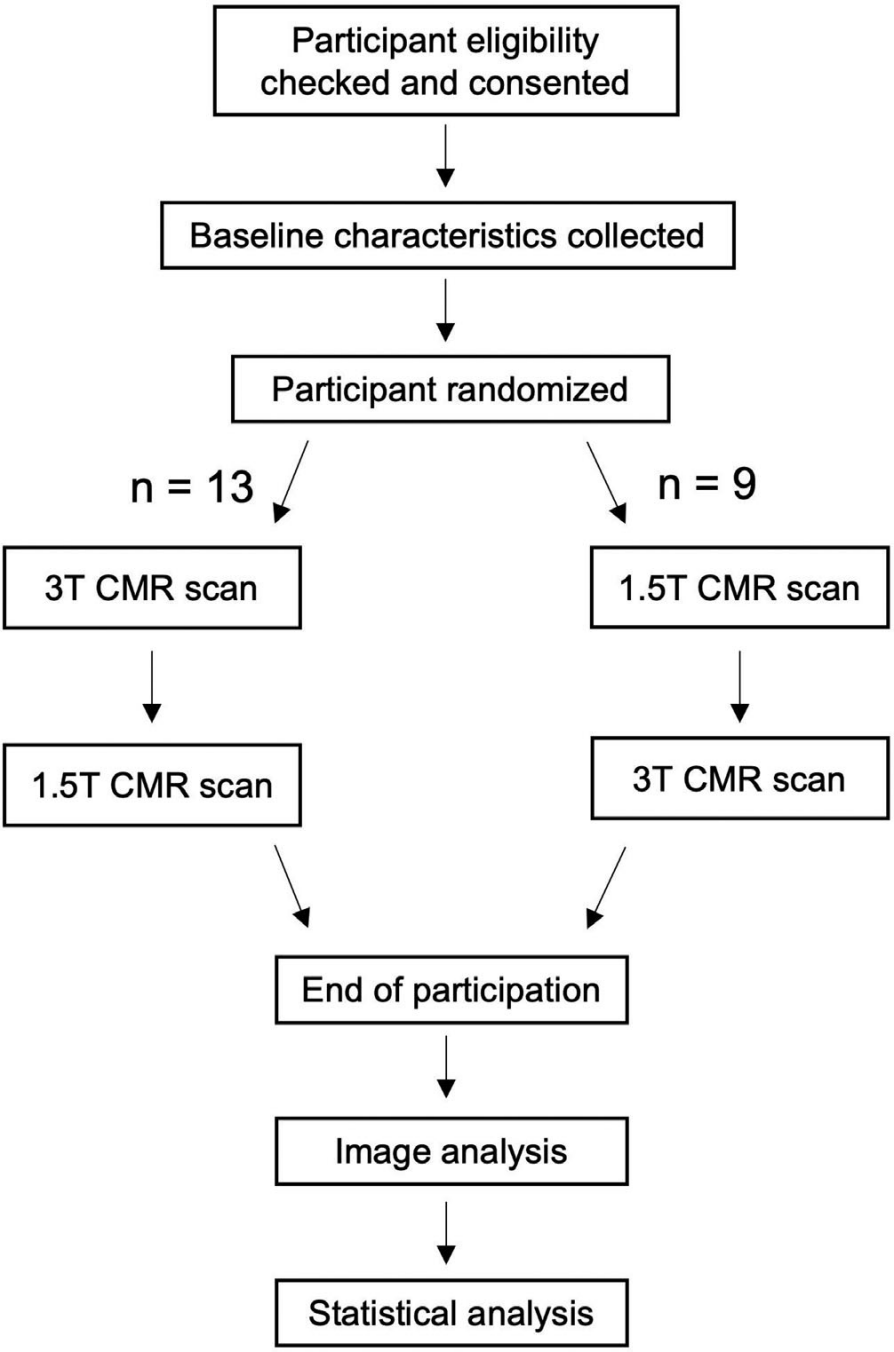
Participant flow through the study.

Subject flow through the study is summarized in Fig. 1. After consent and recruitment into the study, the order of field strength acquisition was randomized using an app‐based random number generator.

### Baseline Measurements

Demographics, medical and medication history were collected. Height and weight were measured for calculation of body mass index (BMI) and body surface area using the Mosteller formula.^27^

### Image Acquisition

Subjects underwent noncontrast cardiac MRI imaging at 3 T (Siemens Skyra, Erlangen, Germany) and 1.5 T (Siemens Aera) within 30 minutes on the same day. All scans were performed by one of two radiographers (K.S.P., J.V.W.; equivalent of radiologic technologists in the United States) and for each participant, the same radiographer performed both scans. Scans were acquired in the supine position with retrospective electrocardiogram gating and an 18‐channel phased‐array cardiac receiver coil. During each scan, short‐ and long‐axis bSSFP cine images were obtained following acquisition of localizers. Short‐axis images ensured coverage of the entire LV. A standardized procedure was used for planning of images at both field strengths using vertical long‐axis, horizontal long‐axis, and short‐axis localizers. Heart rate and blood pressure were recorded during short‐axis and long‐axis cine acquisition. The 8 mm slice thickness and 2 mm gap were used at both field strengths. Cine imaging parameters were field of view = 276 × 340 mm (3 T and 1.5 T), matrix = 208 × 256 (3 T and 1.5 T), voxel size = 1.3 × 1.3 × 8 mm (3 T and 1.5 T), temporal resolution = 48.2 msec (at 3 T) and 46.4 msec (at 1.5 T), repetition time = 3.4 msec (at 3 T) and 2.8 ms (at 1.5 T), and echo times = 1.51 msec (at 3 T) and 1.21 msec (at 1.5 T). All acquisitions were reconstructed to 30 phases, giving a reconstructed temporal resolution range of 23.0–37.7 msec at 1.5 T for heart rate range 53–87 bpm and 22.5–38.5 msec at 3 T for heart rate range 52–89 bpm.

### Image Analysis

All scans were anonymized at the time of acquisition by the radiographer (equivalent of radiologic technologists in the United States). Image analysis commenced only after all participants were recruited and scans were completed. Images were analyzed offline using cvi42 Tissue Tracking (version 5.10, Circle Cardiovascular Imaging, Calgary, Canada) by a single observer (S.L.A., medical doctor with 2 years cardiac MRI experience) and using QStrain (version 2.0, Medis Suite version 3.1, Medis Medical Imaging Systems, Leiden, The Netherlands) by a second observer (A.A., radiographer [equivalent to radiologic technologist] with 4 years of cardiac MRI experience). Both observers were blinded to participant details, order of scanning, field strength, and scanner information (where possible), accepting that differences exist in the appearance of the images at 1.5 T and 3 T such as variations in blood to myocardium contrast and the presence of flow artifacts at 3 T. Short‐ and long‐axis images at both field strengths were assessed separately for image quality by three observers (G.P.M., cardiology consultant with >15 years cardiac MRI experience, SLA, medical doctor with 2 years cardiac MRI experience and AA, radiographer (equivalent to radiologic technologist) with 4 years cardiac MRI experience) on a 4‐point scale; “excellent” when no artifact was present; “very good” some artifact present but not affecting the LV; “fair”: does have artifact, for example, breathing/mis‐triggering, affecting the LV, but images still analyzable and “not analyzable.” The average score of the three observers was then used to describe image quality.

### LV Volumes, Function, and Mass

LV volumes, function, and mass were derived from scans at 1.5 T using cvi42 by manual contouring of the entire short‐axis cine stack, excluding papillary muscles and trabeculations, as previously described.^28^

### LV Strain Analysis With cvi42 Tissue Tracking

**FIGURE 2:**
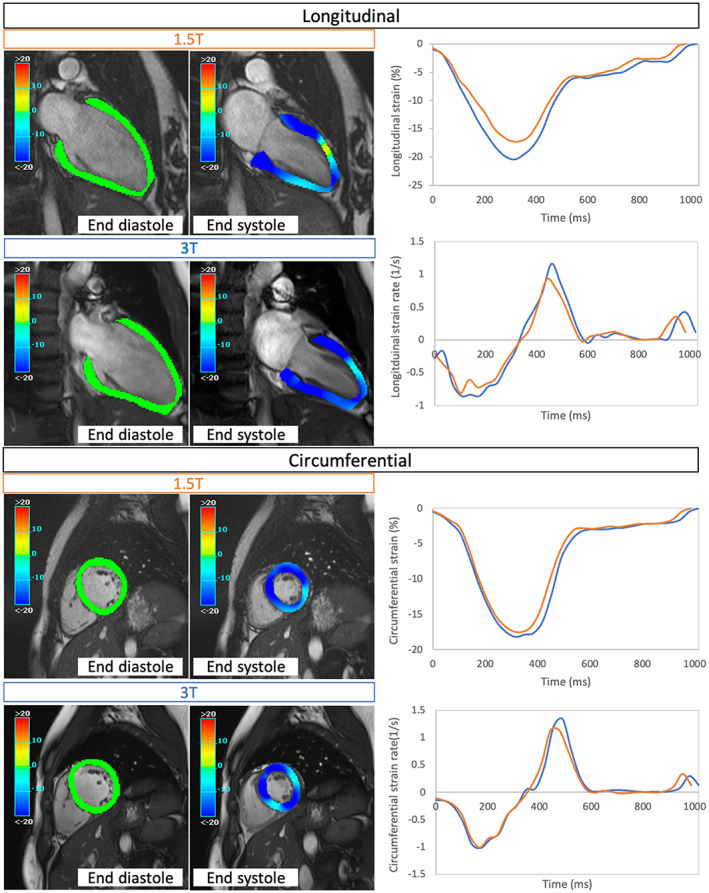
Representative example of image analysis for a single participant with strain and strain rate graphs at 1.5 T (orange) and 3 T (blue) (graphs are presented with negative values for longitudinal and circumferential strain but were transformed to absolute values for analysis and presentation of results).

End‐diastolic epicardial and endocardial borders for long‐axis images were defined manually at 1.5 T and 3 T, as previously described.^17^ Myocardial pixels were then automatically tracked by the software to all phases to generate strain and strain rate measurements. Figure 2 shows a representative example of image analysis for a single participant at both field strengths, alongside strain and strain rate graphs.

GLS and radial strain (long‐axis GRS) were obtained by taking the average of the three long‐axis cine images and GCS and radial strain (short‐axis GRS) from the average of the short‐axis cine images. Peak strain rates (including peak systolic strain rate [PSSR], peak early diastolic strain rate [PEDSR], and peak late diastolic strain rate [PLDSR]) were also measured in the longitudinal, circumferential, and radial (long and short axis) directions, as well as peak torsion. Systolic, early diastolic, and late diastolic peaks were identified from each strain rate curve manually by the observer. The PEDSR and PLDSR were defined as the first and second peaks in strain rate seen in diastole, with the second peak coinciding with atrial contraction. All strain and strain rate values are presented in the results as positive values for ease of interpretation, whereby a lower value represents reduced deformation.

### LV Strain Analysis With Medis QStrain Feature Tracking

Short‐axis endocardial and epicardial borders were manually defined for basal, mid, and apical slices at end‐systolic and end‐diastolic phases, as well as for long‐axis images. Myocardial features were then tracked through the cardiac cycle by the software. Global strain and strain rate measurements (PSSR, PEDSR, and PLDSR) were obtained, as above, by averaging the values for the three short‐axis slices (basal, mid, and apical) and the three long‐axis cine images.

No comparisons between LV strain measurements using cvi42 Tissue Tracking and Medis QStrain were undertaken as marked differences have been demonstrated in absolute strain and strain rate measures with different software vendors.^16^, ^17^, ^21^

### Global Longitudinal Shortening and MAPSE

**FIGURE 3:**
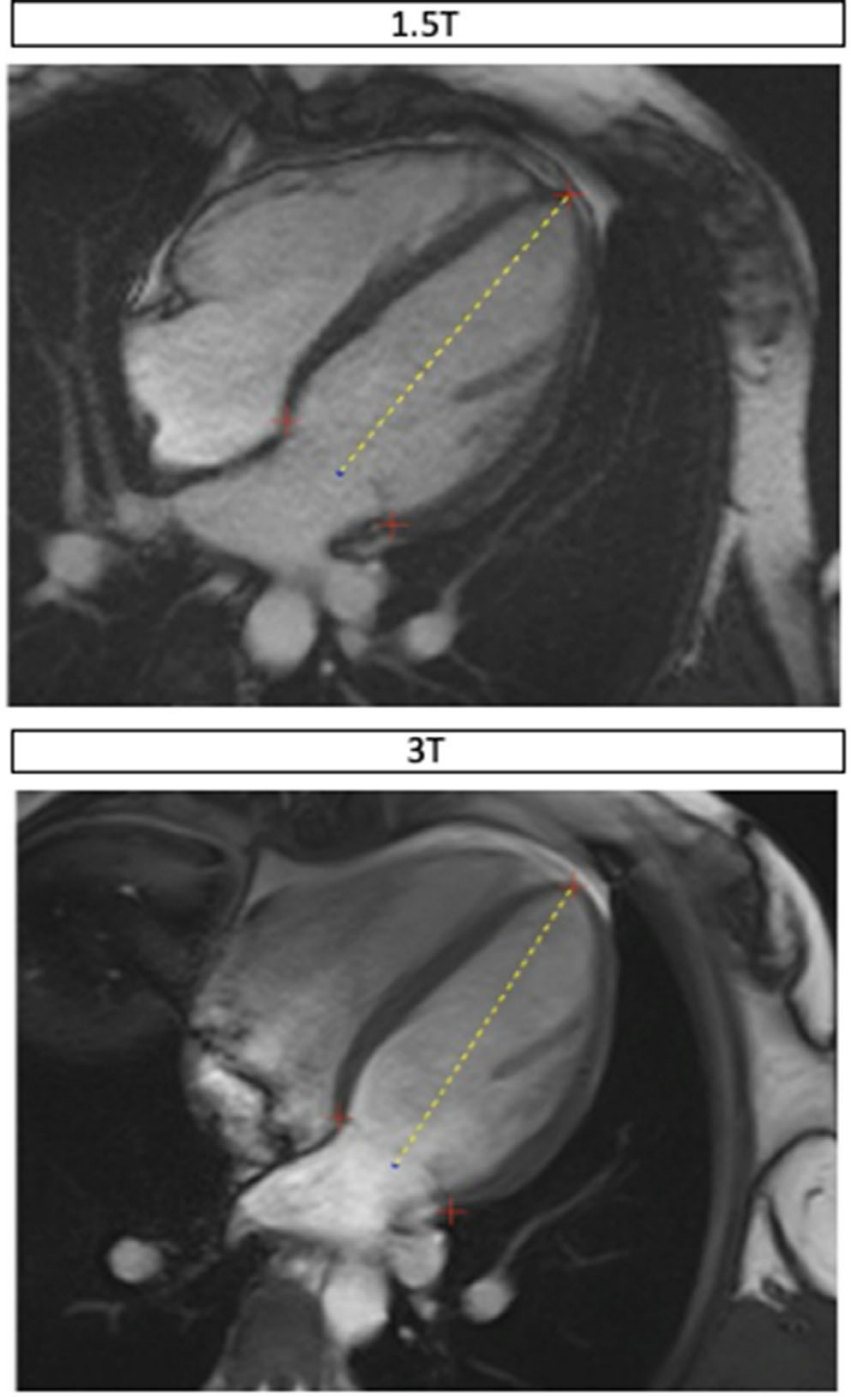
Representative example of image analysis for a single participant at 1.5 T and 3 T for global longitudinal shortening and mitral annular plane systolic excursion (MAPSE). The red crosses represent the mitral annular hinge points and the apex. These are used to measure the length of the left ventricle from the apex to the mid‐point of the valve plane (yellow dashed line).

From long‐axis images, global longitudinal shortening and mitral annular plane systolic excursion (MAPSE) were measured by a fully automated method using an AI deep learning neural network model. The development of this method has been previously described.^18^ Briefly, mitral annular hinge points and apex are marked on all phases of the four‐, three‐, and two‐chamber cine views and this allows measurement of the length of the LV from the apex to the mid‐point of the valve plane (Fig. 3). Global longitudinal shortening was then calculated as the percentage of LV length shortening from end diastole to end systole and MAPSE was the mean moved distance for the two valve points. The mean average was calculated across the three views manually after computation of global longitudinal shortening for each view.

### Interobserver and Intraobserver Variability of LV Strain Analysis With cvi42 Tissue Tracking

The 1.5 T and 3 T scans for 10 randomly selected participants were analyzed using cvi42 Tissue Tracking by a second experienced observer (M.P.M.G., nephrology consultant with 7 years cardiac MRI experience) using manual contours, as described above. The second observer was blinded to subject details and to the results of the first observer. In addition, the first observer (S.L.A.) re‐analyzed scans at both field strengths for 10 different randomly selected participants with a minimum time interval of 4 weeks between analyses to assess intraobserver variability.

### Statistical Analysis

Variables were checked for normality using the Shapiro–Wilk test, histograms, and quantile–quantile plots. Continuous, normally distributed data are presented as mean (standard deviation) and non‐normally distributed data as median (interquartile ranges). Categorical data are presented as frequency (percentage). Differences between hemodynamic (heart rate and blood pressure) and strain measurements were compared between field strengths using Wilcoxon signed rank test. A *P* value < 0.05 was considered statistically significant.

Scatterplots and Spearman's correlations (*r*) were used to assess the correlation of strain measurements at 1.5 T and 3 T. All scatterplots displayed the unity line. To determine the extent of agreement, intraclass correlation coefficients (ICCs) were calculated and Bland–Altman plots were used to identify any systematic bias.^29^ Interpretation of ICCs was based on the following classification: <0.50 poor, 0.50–0.75 fair, 0.75–0.9 good, and ≥0.9 excellent.^30^

A sensitivity analysis was carried out for strain and strain rate measurements using cvi42 Tissue Tracking in subjects with scans rated as excellent or very good at both field strengths, indicating no artifacts within the LV. This included the assessment of correlation and agreement with *r* and ICC values, as well as comparison between field strengths using Wilcoxon signed rank test. Intraobserver and interobserver variability of strain measurements using cvi42 Tissue Tracking were compared with Bland–Altman plots (including bias and limits of agreement generated from these) and ICCs, at each field strength. Differences between observers were also assessed using Wilcoxon signed rank test and correlations for intraobserver and interobserver variability assessed using Spearman's correlation.

Statistical analyses were performed using the R environment for statistical computing (version 4.0.3).^31^

## Results

### Demographics

Baseline characteristics are summarized in Table 1. Mean age was 36 ± 12 years and 45% were male. BMI was 25.3 (22.1, 27.7) kg/m^2^.

**TABLE 1:** Baseline Characteristics and Left Ventricular Volumes, Function, and Mass (at 1.5 T)

Characteristic	*n* = 22
Age (years)	36 ± 12
Gender	
Female	12 (55%)
Male	10 (45%)
Ethnicity	
White British	12 (55%)
Ethnic minority group	10 (45%)
Body mass index (kg/m^2^)	25.3 (22.1, 27.7)
Baseline heart rate (bpm)	75 (69, 81)
Baseline systolic blood pressure (mmHg)	124 ± 17
Baseline diastolic blood pressure (mmHg)	79 ± 11
LV EDV (mL)	153 ± 32
LV EDVi (mL/m^2^)	83 ± 12
LV ESV (mL)	57 ± 15
LV ESVi (mL/m^2^)	31 ± 7
LV SV (mL)	96 ± 21
LV CO (liter/min)	6 ± 2
LV EF (%)	63 ± 6
LVM (g)	93 ± 31
LVMi (g/m^2^)	50 ± 13

Median (IQR), Median ± SD or *n* (%).

CO = cardiac output; EDV = end diastolic volume; EDVi = end diastolic volume indexed to body surface area; EF = ejection fraction; ESV = end systolic volume; ESVi = end systolic volume indexed to body surface area; LV = left ventricular; LVM = left ventricular mass; LVMi = left ventricular mass indexed to body surface area; SV = stroke volume.

### Hemodynamic Measurements During Scan Acquisition

Twenty (91%) participants had full hemodynamic measurements taken during scanning. There were no significant differences in heart rate and blood pressure measurements between 1.5 T and 3 T scans, as shown in Table 2.

**TABLE 2:** Hemodynamic Measurements During Scan Acquisition at 1.5 T and 3 T

	1.5 T	3 T	*P* value
During long‐axis acquisition			
Heart rate (bpm)	64 (61, 72)	66 (59, 70)	0.28
Systolic BP (mmHg)	112 (108, 126)	112 (104, 134)	0.86
Diastolic BP (mmHg)	74 (70, 82)	76 (67, 86)	0.62
During short‐axis acquisition			
Heart rate (bpm)	66 (60, 72)	67 (60, 72)	0.81
Systolic BP (mmHg)	116 (105, 122)	116 (104, 124)	0.61
Diastolic BP (mmHg)	72 (66, 83)	74 (68, 83)	>0.99

Median (IQR).

BP = blood pressure.

### Image Quality

All cardiac MRI images at both field strengths were analyzable. Image quality was rated as good or excellent for all 1.5 T scans, except the long‐axis images for one participant (long‐axis mean image quality score = 2.9 and short‐axis mean image quality score = 2.8). However, image quality was lower at 3 T with a mean of 2.0 for both short‐ and long‐axis images and 4 out of 22 scans for both long‐ and short‐axis images were rated as fair.

### LV Volumes, Function, and Mass

LV volumes and function measurements at 1.5 T are presented in Table 1. All participants had a visually normal scan with no regional wall motion abnormality or LV hypertrophy. Mean LV ejection fraction was 63% ± 6% and all participants had an LV ejection fraction >50%.

### Interfield Strength Agreement of Global LV Strain and Strain Rate Measurements

**FIGURE 4:**
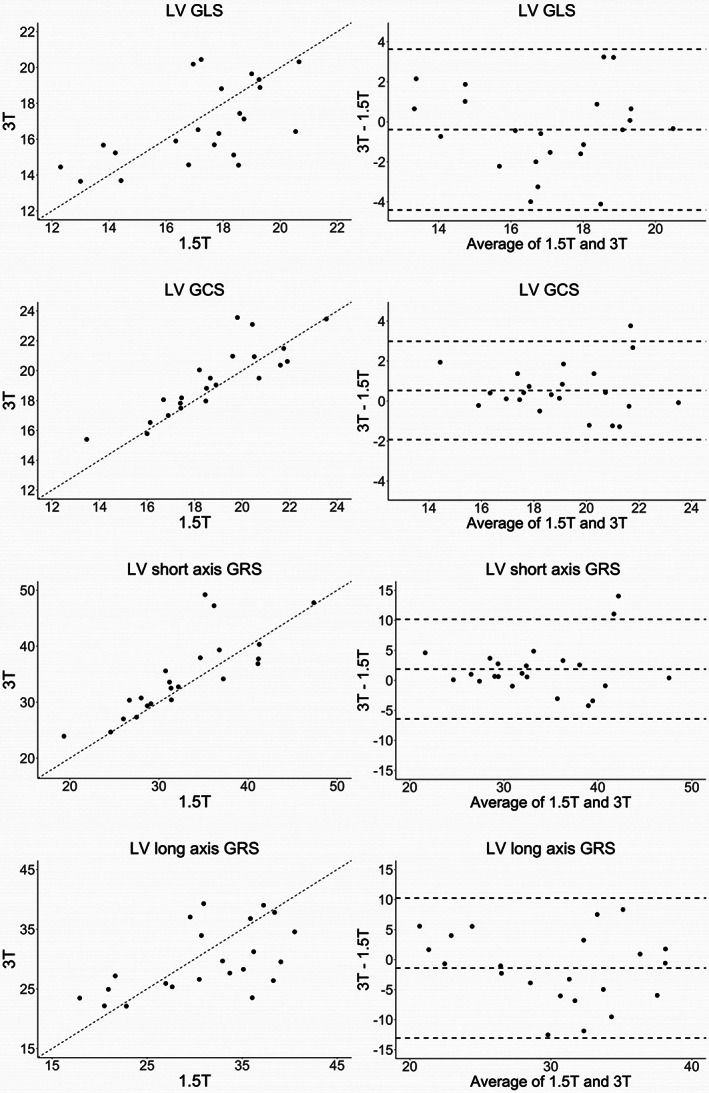
Interfield strength agreement of left ventricular global strain measurements using cvi42 Tissue Tracking. Scatterplots are shown on the left with the unity line (dashed line, *x* = *y*) and Bland Altman plots on the right. GCS = global circumferential strain; GLS = global longitudinal strain; GRS = global radial strain; LV = left ventricular.

Global LV strain, PSSR, PEDSR, PLDSR, and peak torsion measurements at 1.5 T and 3 T for cvi42 Tissue Tracking are displayed in Table 3, along with corresponding *P* values, correlation coefficients (*r*), and ICCs for interfield strength agreement. The same results for Medis QStrain feature tracking are presented in Additional file S1. Figure 4 displays the scatterplots and Bland Altman plots for systolic strain measurements using cvi42 Tissue Tracking. Scatterplots and Bland Altman plots for PSSR, PEDSR, PLDSR and peak torsion using cvi42 Tissue Tracking are presented in Additional file S2 and for all strain and strain rate measurements using Medis QStrain are presented in Additional file S3.

**TABLE 3:** Interfield Strength Agreement of Left Ventricular Strain and Strain Rate Measurements Using Cardiac MRI cvi42 Tissue Tracking at 1.5 T and 3 T

	1.5 T	3 T	*P* value	*r*	ICC
LV GLS (%)	17.8 (16.5, 18.7)	16.4 (15.2, 18.9)	0.41	0.62	0.60
LV GCS (%)	18.6 (17.4, 20.5)	19.3 (17.9, 20.9)	0.06	0.88	0.84
Short‐axis GRS (%)	31.4 (28.2, 36.6)	33.2 (29.9, 37.9)	0.05	0.88	0.79
Long‐axis GRS (%)	31.9 (27.1, 36.2)	28.0 (25.5, 34.4)	0.35	0.59	0.55
Longitudinal PSSR (1/sec)	0.88 (0.77, 0.95)	0.84 (0.71, 0.98)	0.66	0.50	0.48
Circumferential PSSR (1/sec)	0.97 (0.89, 1.04)	0.97 (0.91, 1.08)	0.28	0.84	0.86
Short‐axis radial PSSR (1/sec)	1.55 (1.44, 2.02)	1.54 (1.44, 1.91)	0.94	0.75	0.78
Long‐axis radial PSSR (1/sec)	1.66 (1.28, 1.80)	1.59 (1.28, 1.83)	0.57	0.46	0.39
Longitudinal PEDSR (1/sec)	0.93 (0.75, 1.08)	0.85 (0.65, 1.05)	0.31	0.56	0.64
Circumferential PEDSR (1/sec)	1.16 (1.01, 1.32)	1.21 (1.02, 1.43)	0.03	0.87	0.82
Short‐axis radial PEDSR (1/sec)	2.20 (1.93, 2.85)	2.24 (1.74, 2.71)	0.48	0.76	0.83
Long‐axis radial PEDSR (1/sec)	2.16 (1.53, 2.47)	1.85 (1.41, 2.20)	0.34	0.60	0.61
Longitudinal PLDSR (1/s)	0.49 (0.42, 0.63)	0.50 (0.36, 0.59)	0.03	0.74	0.64
Circumferential PLDSR (1/sec)	0.41 (0.32, 0.49)	0.46 (0.34, 0.51)	0.13	0.87	0.89
Short‐axis radial PLDSR (1/sec)	0.45 (0.35, 0.55)	0.51 (0.37, 0.57)	0.09	0.87	0.91
Long‐axis radial PLDSR (1/sec)	0.54 (0.46, 0.70)	0.56 (0.40, 0.67)	0.06	0.79	0.71
Peak torsion (deg/cm)	1.43 (0.98, 1.75)	1.22 (0.96, 1.64)	0.44	0.31	0.28

Median (IQR).

GCS = global circumferential strain; GLS = global longitudinal strain; GRS = global radial strain; ICC = intraclass correlation co‐efficient; PEDSR = peak early diastolic strain rate; PLDSR = peak late diastolic strain rate; PSSR = peak systolic strain rate; *r* = Spearman's correlation co‐efficient.

Using cvi42 Tissue Tracking, median GLS at 1.5 T was 17.8% (16.5, 18.7) and 16.4% (15.2, 18.9) at 3 T and GCS was 18.6% (17.4, 20.5) and 19.3% (17.9, 20.9), respectively (*P* = 0.41 for GLS, *P* = 0.06 for GCS). Strain and strain rate values derived from long‐axis images (longitudinal and long‐axis radial) showed poor‐to‐fair agreement (ICC range 0.39–0.71), whereas values derived from short‐axis images (circumferential and short‐axis radial) showed good‐to‐excellent agreement between field strengths (ICC range 0.78–0.91). Minimal bias was seen in all systolic strain measurements, as well as strain rate measurements. Peak torsion showed very poor agreement between field strengths (ICC = 0.28).

Using Medis QStrain, short‐axis derived strain and strain rate values showed better interfield agreement (ICC range 0.62–0.90) compared to long‐axis derived images (ICC range 0.31–0.76), although this difference was less pronounced compared to the results with cvi42 Tissue Tracking.

### Sensitivity Analysis by Image Quality

A sensitivity analysis using only images that had no artifact present within the LV (images rated as excellent and very good, long axis and short axis *n* = 18) was undertaken. Minimal differences in correlation co‐efficients and ICC values were seen compared to the complete analysis for all strain and strain rates values, as well as peak torsion (Supplementary Table 2 in Additional file S2).

### Interfield Strength Agreement of Global Longitudinal Shortening and MAPSE

**FIGURE 5:**
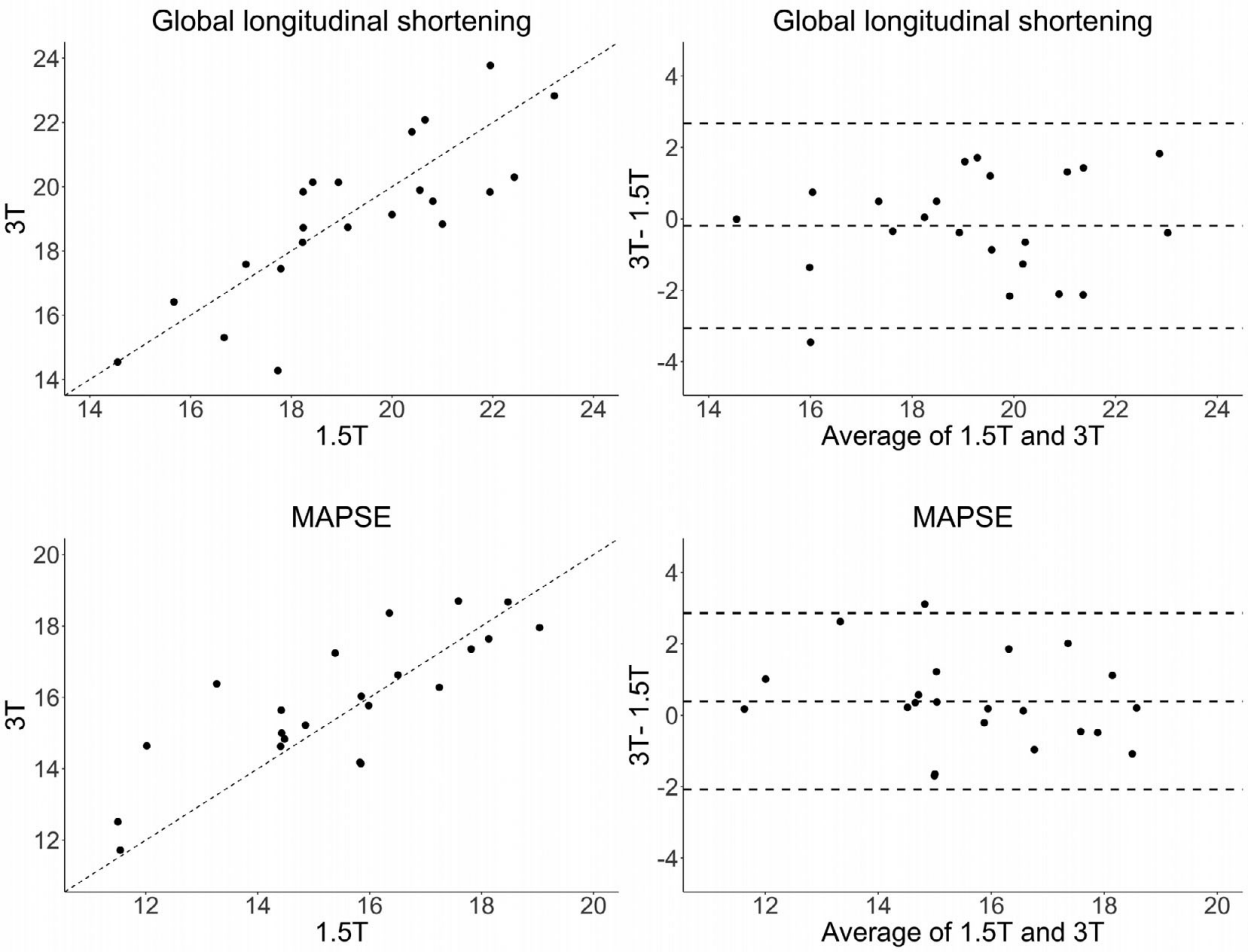
Interfield strength agreement of left ventricular global longitudinal shortening (%) and MAPSE (mm). Scatterplots are shown on the left with the unity line (dashed line, *x* = *y*) and Bland–Altman plots on the right. MAPSE = mitral annular plane systolic excursion.

Global longitudinal shortening was 19.0% (17.9, 20.8) at 1.5 T and 19.3% (17.8, 20.1) at 3 T and MAPSE was 15.8 mm (14.4, 17.1) and 15.9 mm (14.7, 17.3), respectively (*P* = 0.70 and 0.19). Figure 5 displays the scatterplots (with unity lines) and Bland Altman plots for both measurements. Both showed minimal bias and good interfield strength agreement (ICC = 0.81 and 0.80, respectively). Global longitudinal shortening showed tighter limits of agreement compared to LV GLS. Global longitudinal shortening and LV GLS were well correlated at 1.5 T and 3 T (*r* = 0.84 and 0.80, respectively).

### Intraobserver and Interobserver Variability Using cvi42 Tissue Tracking

Supplementary tables 3 and 4 in Additional file S5 show the bias and limits of agreement with ICCs, as well as correlation co‐efficients and *P* values for intraobserver and interobserver variability at each field strength. Supplementary Figures S9–S18 show Bland Altman plots for intraobserver and interobserver variability, respectively, at 1.5 T and 3 T for all measures of strain, strain rates, and peak torsion. Intraobserver variability of strain and strain rate was good to excellent (ICC > 0.75) at both field strengths, except for long‐axis radial PSSR at 3 T (ICC = 0.61) and peak torsion (ICC = 0.62 at 1.5 T and ICC = 0.57 at 3 T), which showed fair agreement between observations. There was minimal bias for systolic strain measurements, except with long‐axis derived radial strain, which, at both field strengths, showed a bias of −2. All strain and strain rate values derived from short‐axis images (circumferential and short‐axis radial) showed good‐to‐excellent interobserver variability, at both field strengths (ICC > 0.75). However, there was a bias toward higher LV GCS and short‐axis derived GRS values for observer 2. Values derived from long‐axis images (longitudinal and long‐axis radial) showed better interobserver variability at 1.5 T compared to 3 T, with the exception of long‐axis radial PSSR (ICC = 0.73 at 3 T and ICC = 0.85 at 3 T). Overall, at both 1.5 T and 3 T, intraobserver variability for all values was slightly better compared to the interfield strength agreement, as was interobserver variability for short‐axis derived values. Interobserver variability at 3 T for long‐axis images, however, showed similar results to interfield strength agreement for strain and strain rate values.

## Discussion

In this prospective randomized cross‐sectional study of healthy volunteers, the interfield strength agreement of LV strain and strain rates was good to excellent for measurements derived from short‐axis images, but poor to fair for measurements from long‐axis images. There was minimal bias between field strengths for all measurements. Similar results for the interfield strength agreement of strain and strain rate measurement were found using two software vendors and when considering only images with no artifacts present within the LV. AI‐derived global longitudinal shortening and MAPSE showed good agreement.

Previous studies, which have compared strain measurements using bSSFP cine‐based techniques at 1.5 T and 3 T, have used two separate groups of individuals and therefore, their subsequent results do not represent a direct comparison of interfield strength agreement.^25^, ^32^, ^33^ This study describes interfield strength agreement of strain measurements derived from cardiac MRI bSSFP cine images acquired in the same subjects at both 1.5 T and 3 T. In a previous work using displacement‐encoding with stimulated echoes in 89 participants scanned 1.4 days apart, GLS has been found to be greater at 1.5 T compared to 3 T with similar results for circumferential strain on a segmental level.^34^ Our results show minimal bias of strain and strain rate measurements derived from bSSFP cine images.

The results of this work suggest that interfield strength agreement is better for strain values derived from short‐axis images compared to long‐axis images. These results are similar to previous studies that have assessed the test–retest reproducibility of strain measurements, whereby values derived from short‐axis images have better reproducibility compared to long‐axis values, particularly for diastolic strain rates.^14^, ^35^ The reason for these differences is not clear but may represent poorer tracking of the myocardial/blood pool border for long‐axis images, especially at the apex. Additionally, despite careful planning of images between scanners using the same localizer acquisition, even small differences in slice position may impact results from long‐axis images more readily compared to short‐axis images. However, the test–retest reproducibility of LV GLS at both 1.5 T and 3 T appears to be better than the interfield strength agreement found in this study, suggesting the differences in field strength of the two scans are contributing to the differences seen in long‐axis strain measurements.^17^

The findings of this study could represent a difference in image quality between 1.5 T and 3 T. Poor image quality may affect the ability for software to track pixels through the cardiac cycle and could affect the derived strain and strain values. Higher field strengths are more susceptible to artifact, and in this study, artifact in the region of interest was only present in scans performed at 3 T (four participants in total). However, despite this, the scans remained analyzable using both software vendors. The sensitivity analysis performed, which excluded participants with scans containing artifact in the LV, did not change the agreement in strain and strain rates. This suggests that the differences seen between field strengths are unlikely to be explained by differences in image quality.

Global longitudinal shortening may be less likely to be affected by the potential issues seen with strain derived from long‐axis images and this may be why better interfield strength agreement was demonstrated in this study compared to LV GLS, despite both measurements being derived from the same images. With similarities to our findings, intraobserver and interobserver reproducibility for global longitudinal shortening using a semi‐automated method has previously been shown to be better compared to GLS by feature tracking in a cohort consisting of normal controls and patients with heart failure.^19^

As stress (exercise or pharmacological) increases LV GLS in healthy controls,^36^, ^37^ we randomized participants to field strength to minimize any systematic impact of anxiety on hemodynamic measurements. Noninvasive heart rate and blood pressure parameters were systematically measured during scan acquisition and were not different between participants at 1.5 T and 3 T.

Prior studies of healthy volunteers have mainly used scans at 1.5 T. Only one study at 3 T was included in a meta‐analysis of strain measurements derived from bSSFP cine images.^22^ The reported pooled means for median GLS and GCS from this meta‐analysis were higher compared to our results for both field strengths using cvi42 Tissue Tracking; however, different software vendors were used to this in most of the studies included. Our values for GLS (17.8% and 16.4% at 1.5 T and 3 T, respectively) are similar to other investigators' previously published normal value using cvi42 Tissue Tracking in controls scanned at either 1.5 T or 3 T.^33^, ^38^ The strain and strain rate values, with the exception of PLDSR, were higher in our analysis using Medis QStrain and this was most notable for GRS. These differences are consistent with previous studies assessing intervendor reproducibility.^16^, ^21^ It is important to recognize that different software packages provide different strain values from the same cine images, due to differences in analysis methods. Similar results for interfield strength agreement were seen using both software vendors, suggesting the differences seen in long‐axis derived values between 1.5 T and 3 T are not software specific. Further assessment with other software vendors and strain analysis techniques are, however, needed.

A major strength of this study is that the same individuals were scanned at both field strengths on the same day. Importantly, the order of scanning was randomized to minimize bias and no differences in hemodynamic measurements were seen between field strengths. Participants were transferred to the second scanner immediately following the first acquisition and were scanned by the same radiographer at both field strengths, further minimizing acquisition bias. In addition, the analysis was undertaken using two different software vendors, providing evidence that the results are not software specific and this increases the robustness of the results.

The results of this study have two potentially important implications. 1) For participants in interventional or longitudinal research studies where strain measurements are repeated it is important that acquisitions are performed at the same field strength (and ideally the same scanner pending further studies). Between group comparisons should take field strength into consideration: that 1.5 T and 3 T results are not interchangeable, particularly for longitudinal strain. 2) In clinical practice, it is likely that specific normal ranges are needed for both 1.5 T and 3 T, and these should be vendor and technique specific given the results of previous studies.^14^, ^15^, ^17^

### Limitations

The sample size of this study is relatively small, but similar to other studies that have assessed agreement and interstudy reproducibility.^25^, ^32^, ^35^ These results apply to two specific Siemen's scanners in a single center and two vendor's analysis software and therefore cannot be generalized more widely. Furthermore, this study was performed in healthy volunteers and there may be differences seen in the interfield strength agreement of strain in diseased populations. With impaired strain and strain rate measurements tracking of the myocardium may be more difficult and therefore we could postulate that similar or even bigger differences between field strengths will be seen in diseased populations. Further study is required to assess this.

### Conclusions

The interfield strength agreement of short‐axis derived LV strain and strain rate measurements at 1.5 T and 3 T was better than long‐axis derived measurements. However, the agreement of AI landmark‐based global longitudinal shortening and MAPSE was good. These findings have implications for both clinical and research MRI studies, which quantify myocardial strain.

## Supporting information


Additional file 1 Title and description of data:
Supplementary Table 1: Inter‐field strength agreement of left ventricular strain and strain rate measurements using QStrain at 1.5 T and 3 T. Intraclass correlation co‐efficients (ICCs) and Spearman's correlation co‐efficients (r) included.

Additional file 2 Title and description of data:
Supplementary figure 1: Inter‐field strength agreement of LV PSSR using cvi42
LAx, long axis; LV, left ventricular; PSSR, peak systolic strain rate; SAx, short axis
Supplementary figure 2: Inter‐field strength agreement of LV PEDSR using cvi42
LAx, long axis; LV, left ventricular; PEDSR, peak early diastolic strain rate; SAx, short axis
Supplementary figure 3: Inter‐field strength agreement of LV PLDSR using cvi42
LAx, long axis; LV, left ventricular; PLDSR, peak late diastolic strain rate; SAx, short axis
Supplementary figure 4: Inter‐field strength agreement of LV peak torsion using cvi42
LV, left ventricular

Additional file 3 Title and description of data:
Supplementary figure 5: Inter‐field strength agreement of LV strain using QStrain
GCS, global circumferential strain; GLS, global longitudinal strain; GRS, global radial strain; LAx, long axis; LV, left ventricular; SAx, short axis
Supplementary figure 6: Inter‐field strength agreement of LV PSSR using QStrain
LAx, long axis; LV, left ventricular; PSSR, peak systolic strain rate; SAx, short axis
Supplementary figure 7: Inter‐field strength agreement of LV PEDSR using QStrain
LAx, long axis; LV, left ventricular; PEDSR, peak early diastolic strain rate; SAx, short axis
Supplementary figure 8: Inter‐field strength agreement of LV PLDSR using QStrain
LAx, long axis; LV, left ventricular; PLDSR, peak late diastolic strain rate; SAx, short axis

Additional file 4 Title and description of data:
Supplementary Table 2: Sensitivity analysis for the inter‐field strength agreement of left ventricular strain and strain rate measurements using cvi42 Tissue Tracking at 1.5 T and 3 T using excellent and very good image quality scans (n = 18 for long axis images and n = 18 for short axis images)

Additional file 5 Title and description of data:
Supplementary Table 3: Intra‐observer variability of strain and strain rate measurements using cvi42 Tissue Tracking at 1.5 T and 3 T
Supplementary Table 4: Inter‐observer variability of strain and strain rate measurements using cvi42 Tissue Tracking at 1.5 T and 3 T

Additional file 6 Title and description of data:
Supplementary figure 9 Intra‐observer variability of LV global strain
GCS, global circumferential strain; GLS, global longitudinal strain; GRS, global radial strain; LAx, long axis; LV, left ventricular; SAx, short axis
Supplementary figure 10: Intra‐observer variability of LV PSSR
LAx, long axis; LV, left ventricular; PSSR, peak systolic strain rate; SAx, short axis
Supplementary figure 11: Intra‐observer variability of LV PEDSR
LAx, long axis; LV, left ventricular; PEDSR, peak early diastolic strain rate; SAx, short axis
Supplementary figure 12: Intra‐observer variability of LV PLDSR
LAx, long axis; LV, left ventricular; PLDSR, peak late diastolic strain rate; SAx, short axis
Supplementary figure 13: Intra‐observer variability of peak torsion

Additional file 7 Title and description of data:
Supplementary figure 14: Inter‐observer variability of LV global strain
GCS, global circumferential strain; GLS, global longitudinal strain; GRS, global radial strain; LAx, long axis; LV, left ventricular; SAx, short axis
Supplementary figure 15: Inter‐observer variability of LV PSSR
LAx, long axis; LV, left ventricular; PSSR, peak systolic strain rate; SAx, short axis
Supplementary figure 16: Inter‐observer variability of LV PEDSR
LAx, long axis; LV, left ventricular; PEDSR, peak early diastolic strain rate; SAx, short axis
Supplementary figure 17: Inter‐observer variability of LV PLDSR
LAx, long axis; LV, left ventricular; PLDSR, peak late diastolic strain rate; SAx, short axis
Supplementary figure 18: Inter‐observer variability of peak torsion
