# Supplementary material for: The Interfield Strength Agreement of Left Ventricular Strain Measurements at 1.5 T and 3 T Using Cardiac MRI Feature Tracking
Source: J Magn Reson Imaging. 2022 Jun 29;57(4):1250–61. doi: 10.1002/jmri.28328 (PMC10947203; doi:10.1002/jmri.28328)
Supplement: Supplementary file 1 — Additional file 1 Title and description of data: Supplementary Table 1: Inter‐field strength agreement of left ventricular strain and strain rate measurements using QStrain at 1.5 T and 3 T. Intraclass correlation co‐efficients (ICCs) and Spearman's correlation co‐efficients (r) included. [file JMRI-57-1250-s005.pdf]

**Supplementary Table 1:** Inter-field strength agreement of left ventricular strain and strain rate measurements using Medis QStrain at 1.5T and 3T

| | 1.5T | 3T | p value | r | ICC |
|---|---|---|---|---|---|
| LV GLS (%) | 19.2 (18.3, 20.5) | 20.1 (18.8, 21.1) | 0.73 | 0.56 | 0.45 |
| LV GCS (%) | 21.7 (19.7, 24.3) | 21.7 (19.6, 24.3) | 0.80 | 0.87 | 0.87 |
| Short axis GRS (%) | 77.8 (67.9, 88.5) | 83.5 (69.5, 96.5) | 0.31 | 0.75 | 0.62 |
| Long axis GRS (%) | 61.2 (54.3, 68.0) | 60.0 (55.0, 62.5) | 0.52 | 0.16 | 0.34 |
| Longitudinal PSSR (1/s) | 0.92 (0.86, 1.05) | 0.92 (0.84, 1.05) | 0.58 | 0.56 | 0.54 |
| Circumferential PSSR (1/s) | 1.13 (1.05, 1.33) | 1.07 (0.98, 1.23) | 0.23 | 0.89 | 0.80 |
| Short axis radial PSSR (1/s) | 2.42 (2.22, 2.70) | 2.50 (2.22, 3.18) | 0.31 | 0.77 | 0.62 |
| Long axis radial PSSR (1/s) | 1.96 (1.75, 2.10) | 1.94 (1.71, 2.08) | 0.72 | 0.43 | 0.61 |
| Longitudinal PEDSR (1/s) | 1.00 (0.78, 1.16) | 0.96 (0.78, 1.14) | 0.85 | 0.68 | 0.69 |
| Circumferential PEDSR (1/s) | 1.29 (1.09, 1.42) | 1.27 (1.09, 1.60) | 0.27 | 0.77 | 0.65 |
| Short axis radial PEDSR (1/s) | 3.34 (3.09, 3.66) | 3.39 (3.21, 4.53) | 0.071 | 0.70 | 0.69 |
| Long axis radial PEDSR (1/s) | 2.16 (1.94, 2.46) | 2.12 (1.78, 2.42) | 0.70 | 0.62 | 0.75 |
| Longitudinal PLDSR (1/s) | 0.38 (0.30, 0.43) | 0.36 (0.33, 0.44) | 0.56 | 0.33 | 0.31 |
| Circumferential PLDSR (1/s) | 0.28 (0.22, 0.33) | 0.30 (0.25, 0.36) | 0.46 | 0.75 | 0.90 |
| Short axis radial PLDSR (1/s) | 0.26 (0.15, 0.63) | 0.31 (0.11, 0.67) | 0.68 | 0.77 | 0.69 |

| | 1.5T | 3T | p value | r | ICC |
|---|---|---|---|---|---|
| Long axis radial PLDSR (1/s) | 0.43 (0.35, 0.66) | 0.51 (0.35, 0.73) | 0.63 | 0.66 | 0.76 |

Median (IQR)

GCS, global circumferential strain; GLS, global longitudinal strain; GRS, global radial strain; ICC, intraclass correlation co-efficient; PEDSR, peak early diastolic strain rate; PLDSR, peak late diastolic strain rate; PSSR, peak systolic strain rate; r, Spearman's correlation co-efficient
