# Supplementary material for: The Interfield Strength Agreement of Left Ventricular Strain Measurements at 1.5 T and 3 T Using Cardiac MRI Feature Tracking
Source: J Magn Reson Imaging. 2022 Jun 29;57(4):1250–61. doi: 10.1002/jmri.28328 (PMC10947203; doi:10.1002/jmri.28328)
Supplement: Supplementary file 2 — Additional file 2 Title and description of data: Supplementary figure 1: Inter‐field strength agreement of LV PSSR using cvi42 LAx, long axis; LV, left ventricular; PSSR, peak systolic strain rate; SAx, short axis Supplementary figure 2: Inter‐field strength agreement of LV PEDSR using cvi42 LAx, long axis; LV, left ventricular; PEDSR, peak early diastolic strain rate; SAx, short axis Supplementary figure 3: Inter‐field strength agreement of LV PLDSR using cvi42 LAx, long axis; LV, left ventricular; PLDSR, peak late diastolic strain rate; SAx, short axis Supplementary figure 4: Inter‐field strength agreement of LV peak torsion using cvi42 LV, left ventricular [file JMRI-57-1250-s004.pdf]

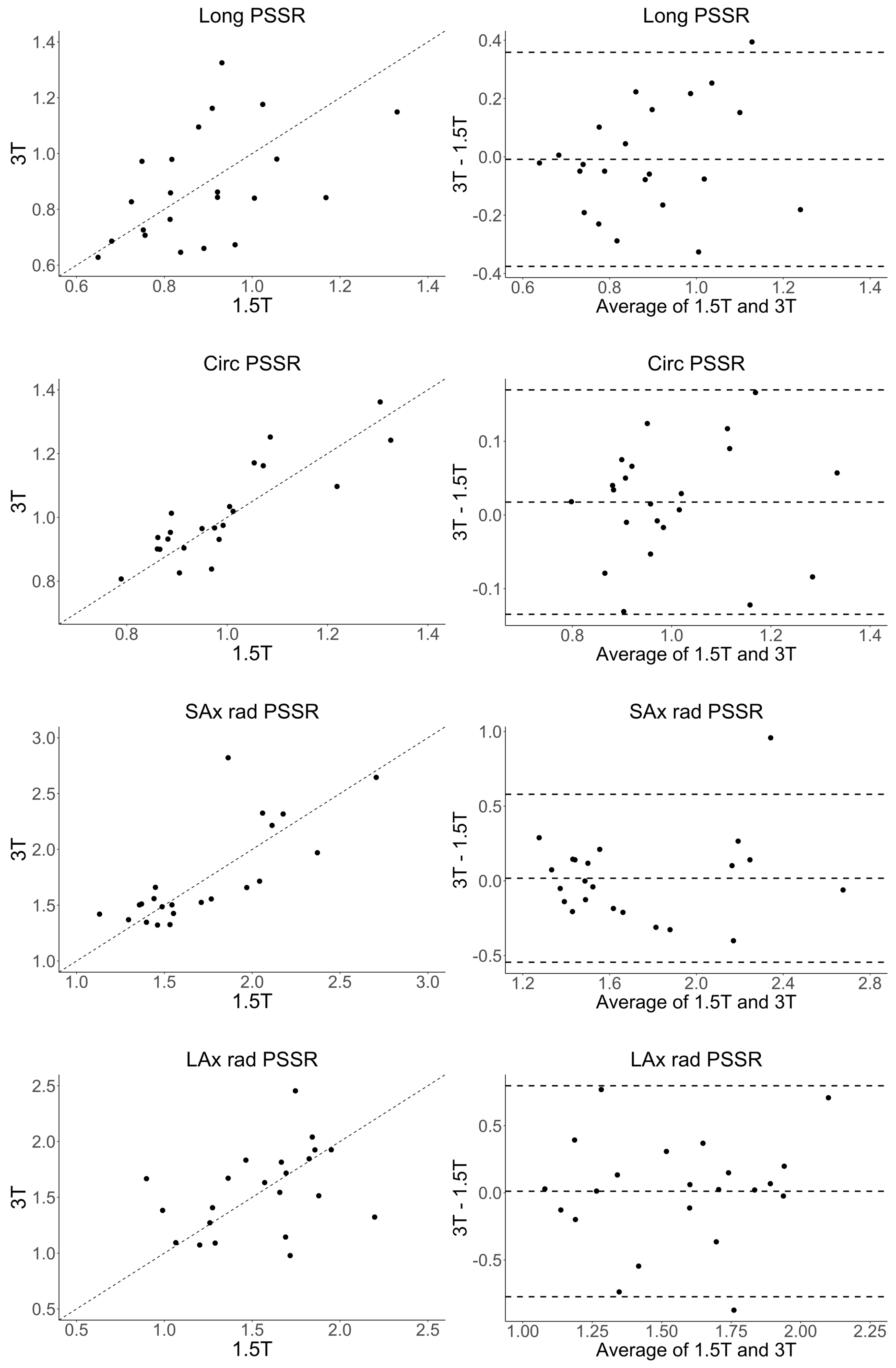

Supplementary figure 1: Inter-field strength agreement of LV PSSR using cvi42

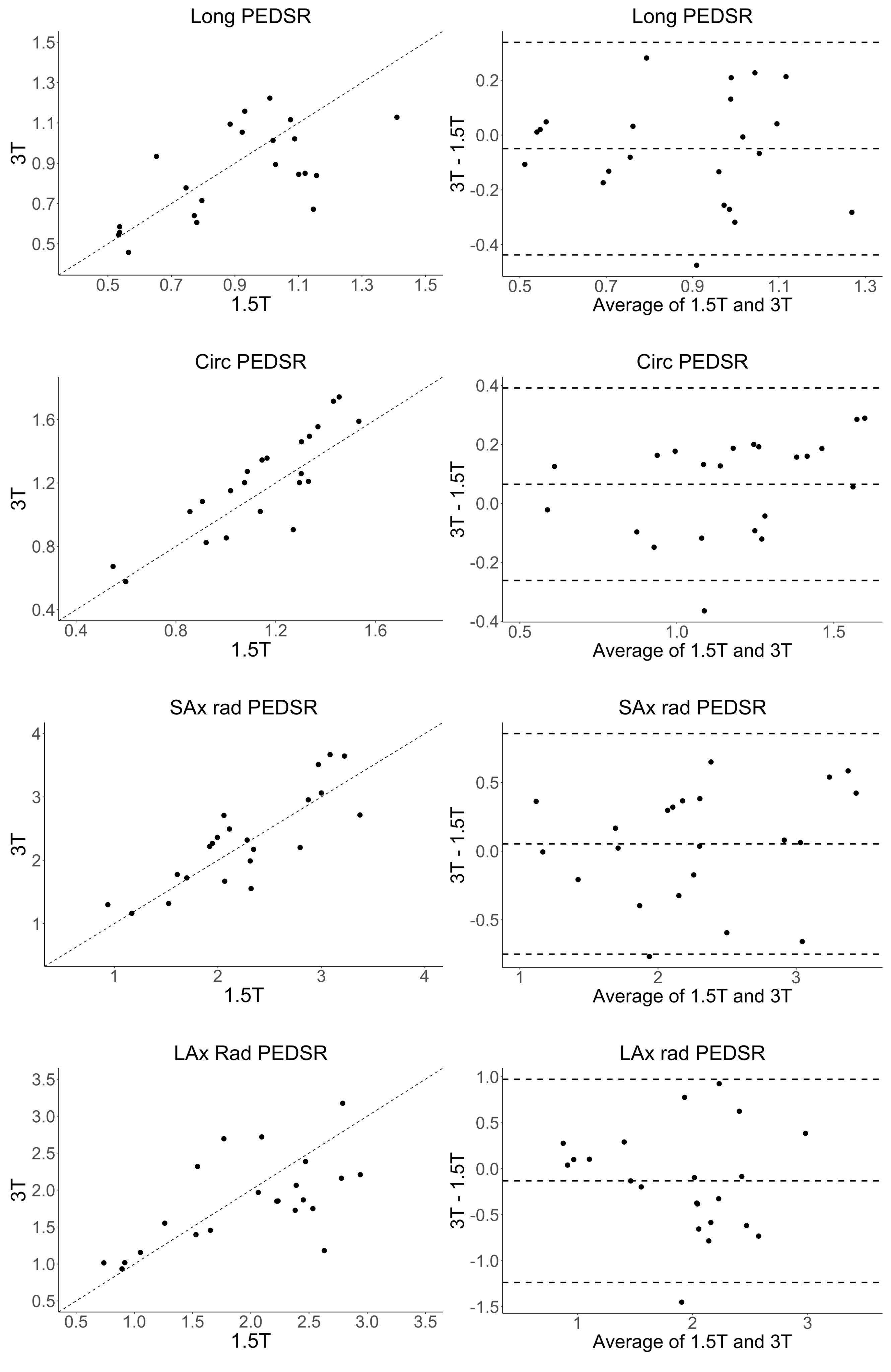

Supplementary figure 2: Inter-field strength agreement of LV PEDSR using cvi42

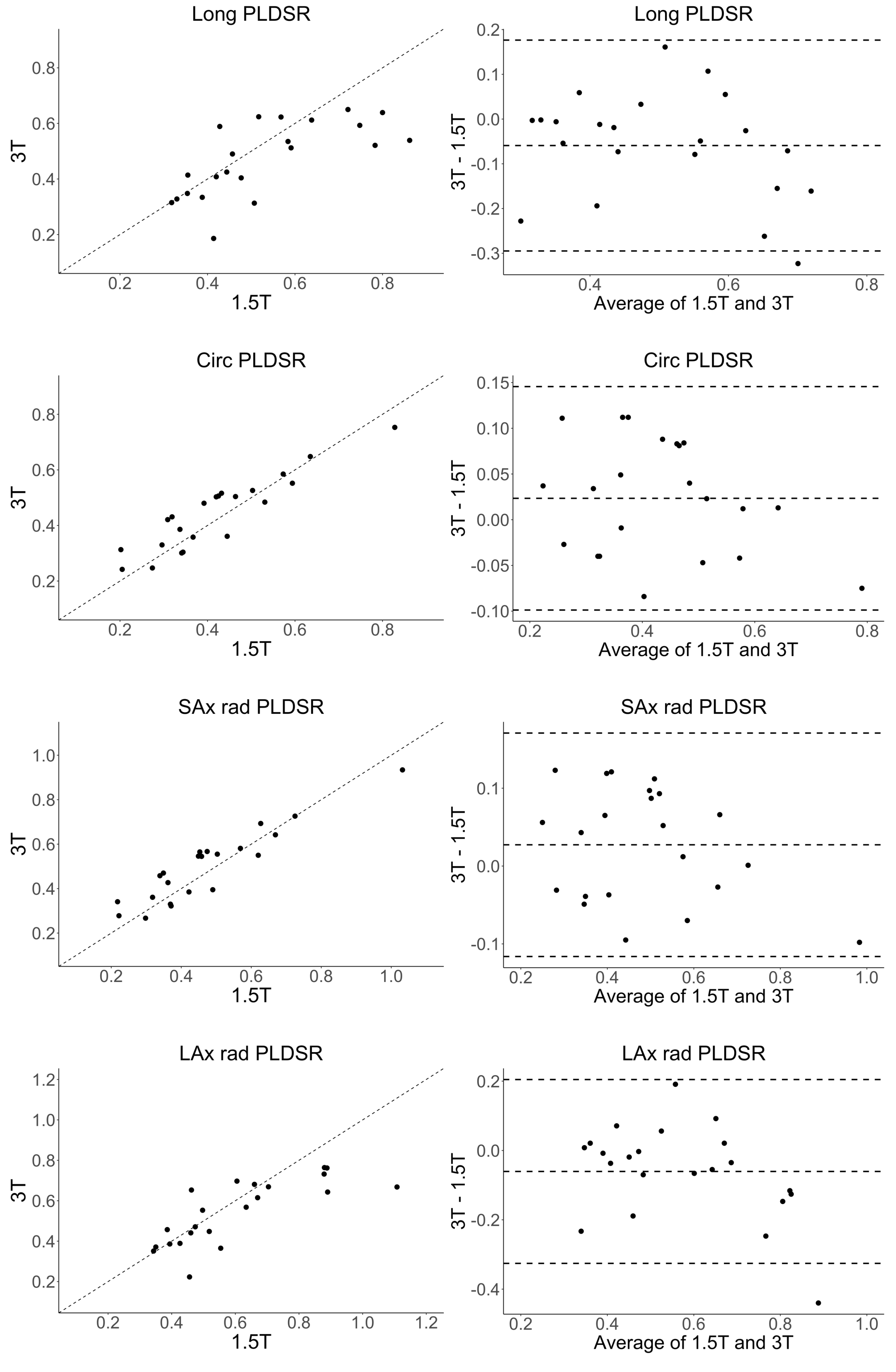

Supplementary figure 3: Inter-field strength agreement of LV PLDSR using cvi42

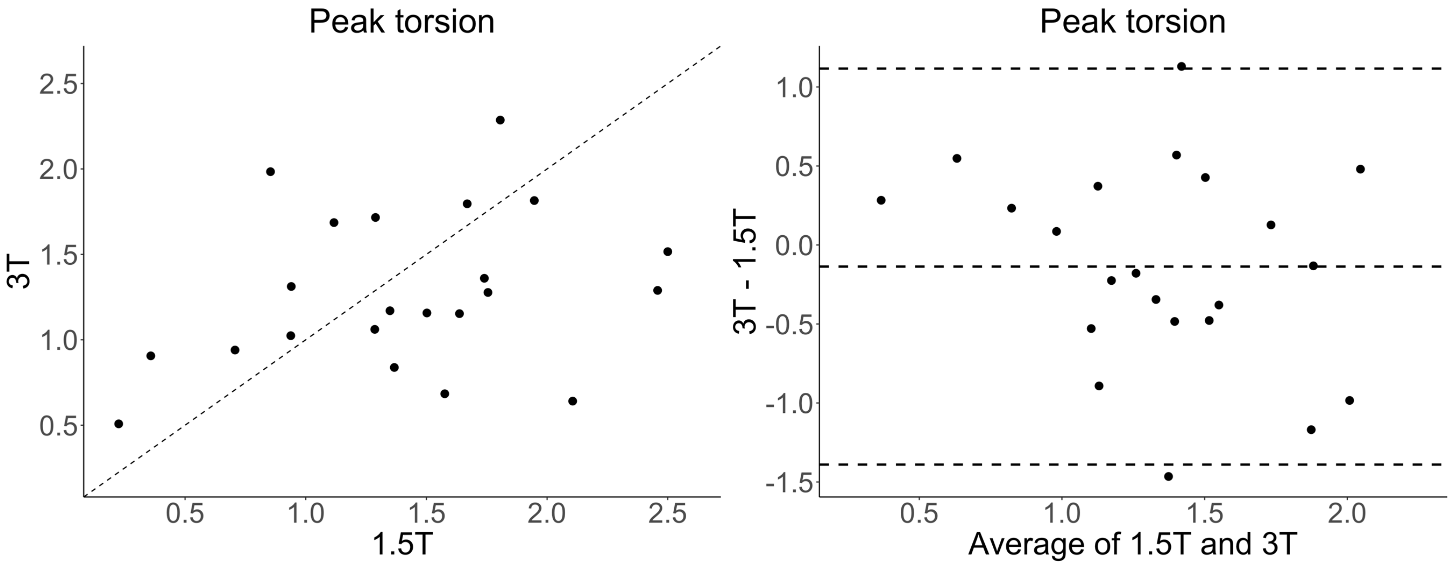

Supplementary figure 4: Inter-field strength agreement of peak torsion using cvi42
