# Supplementary material for: The Interfield Strength Agreement of Left Ventricular Strain Measurements at 1.5 T and 3 T Using Cardiac MRI Feature Tracking
Source: J Magn Reson Imaging. 2022 Jun 29;57(4):1250–61. doi: 10.1002/jmri.28328 (PMC10947203; doi:10.1002/jmri.28328)
Supplement: Supplementary file 3 — Additional file 3 Title and description of data: Supplementary figure 5: Inter‐field strength agreement of LV strain using QStrain GCS, global circumferential strain; GLS, global longitudinal strain; GRS, global radial strain; LAx, long axis; LV, left ventricular; SAx, short axis Supplementary figure 6: Inter‐field strength agreement of LV PSSR using QStrain LAx, long axis; LV, left ventricular; PSSR, peak systolic strain rate; SAx, short axis Supplementary figure 7: Inter‐field strength agreement of LV PEDSR using QStrain LAx, long axis; LV, left ventricular; PEDSR, peak early diastolic strain rate; SAx, short axis Supplementary figure 8: Inter‐field strength agreement of LV PLDSR using QStrain LAx, long axis; LV, left ventricular; PLDSR, peak late diastolic strain rate; SAx, short axis [file JMRI-57-1250-s001.pdf]

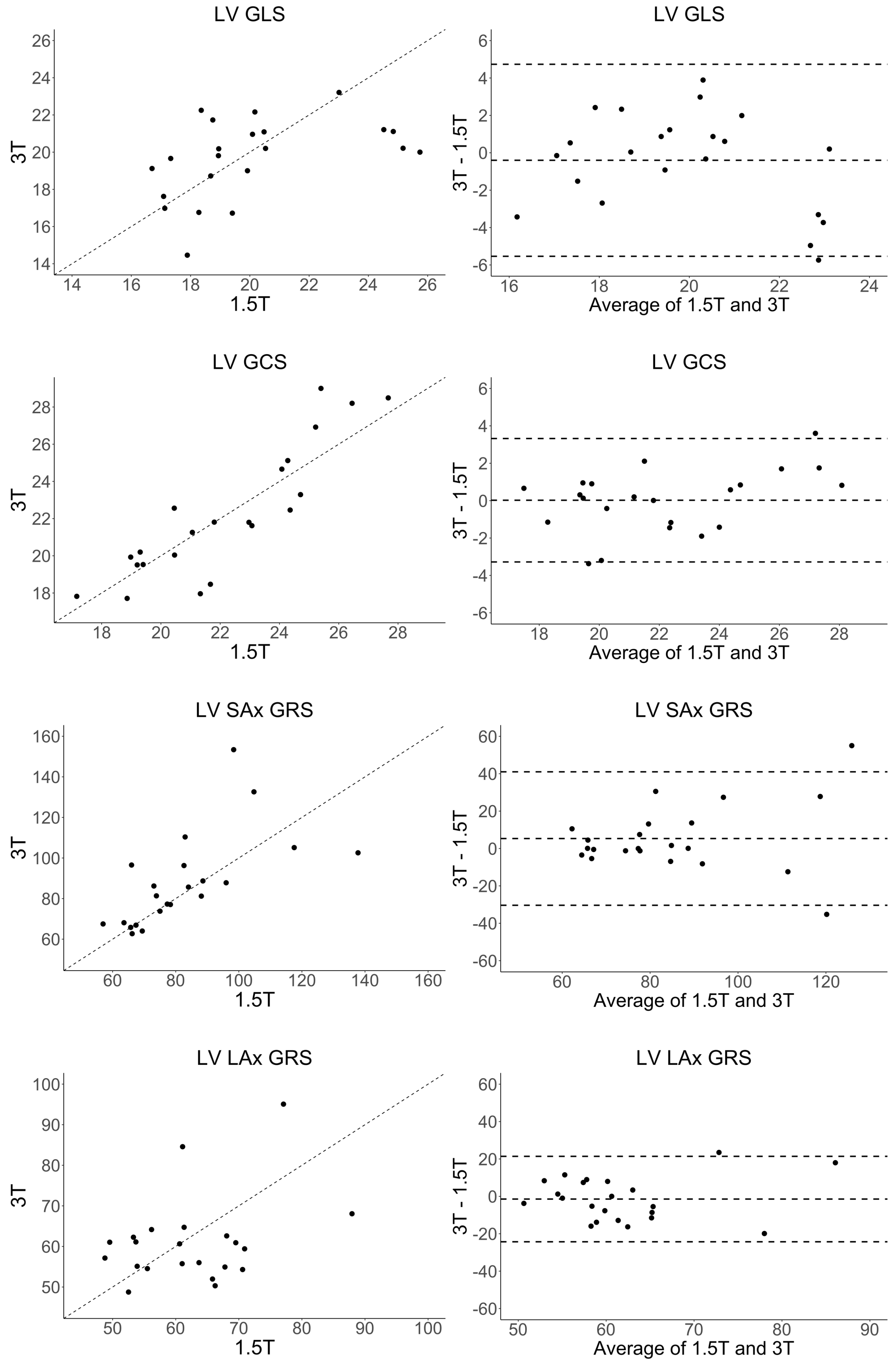

Supplementary figure 5: Inter-field strength agreement of LV strain using QStrain

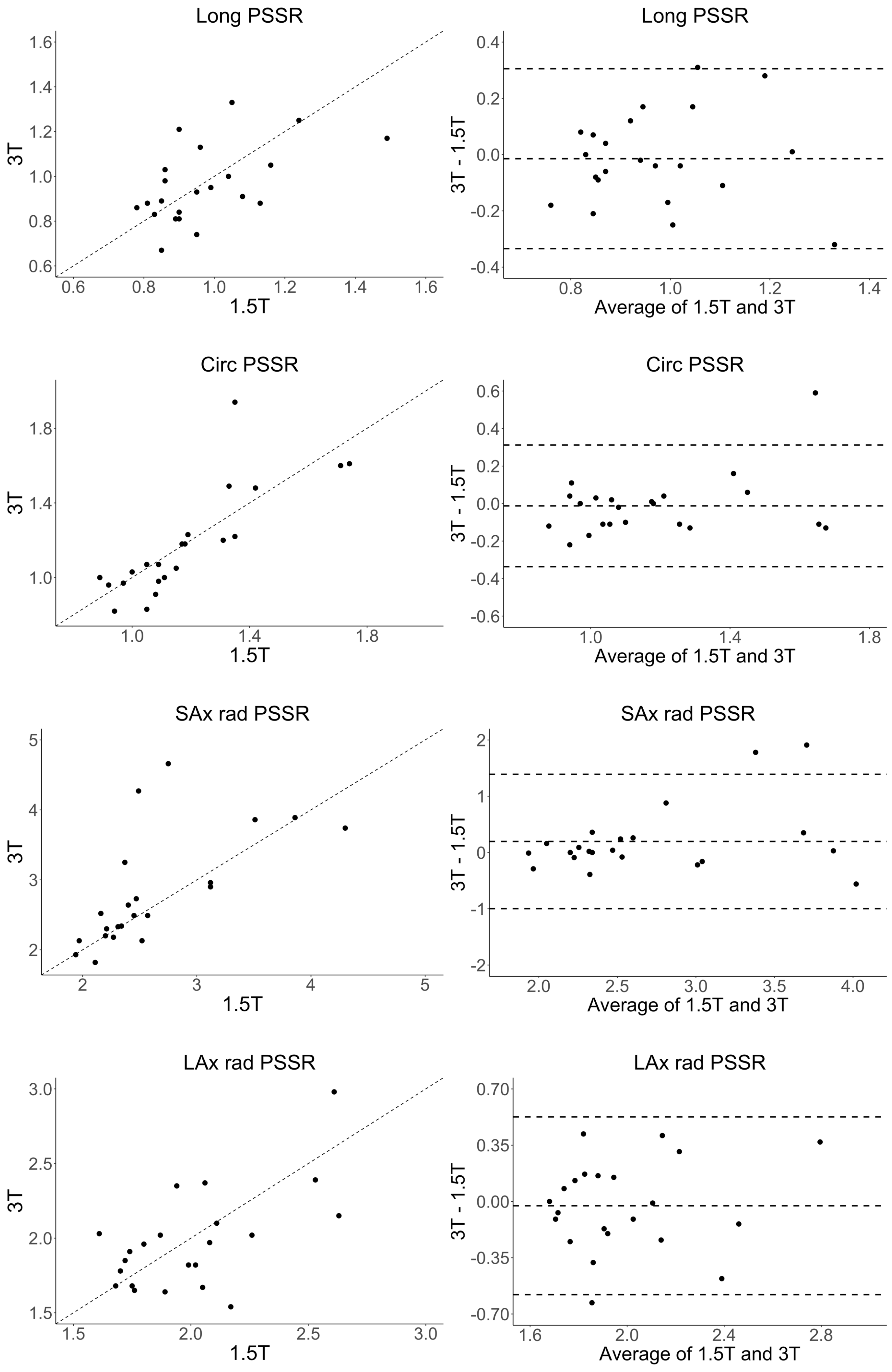

Supplementary figure 6: Inter-field strength agreement of LV PSSR using QStrain

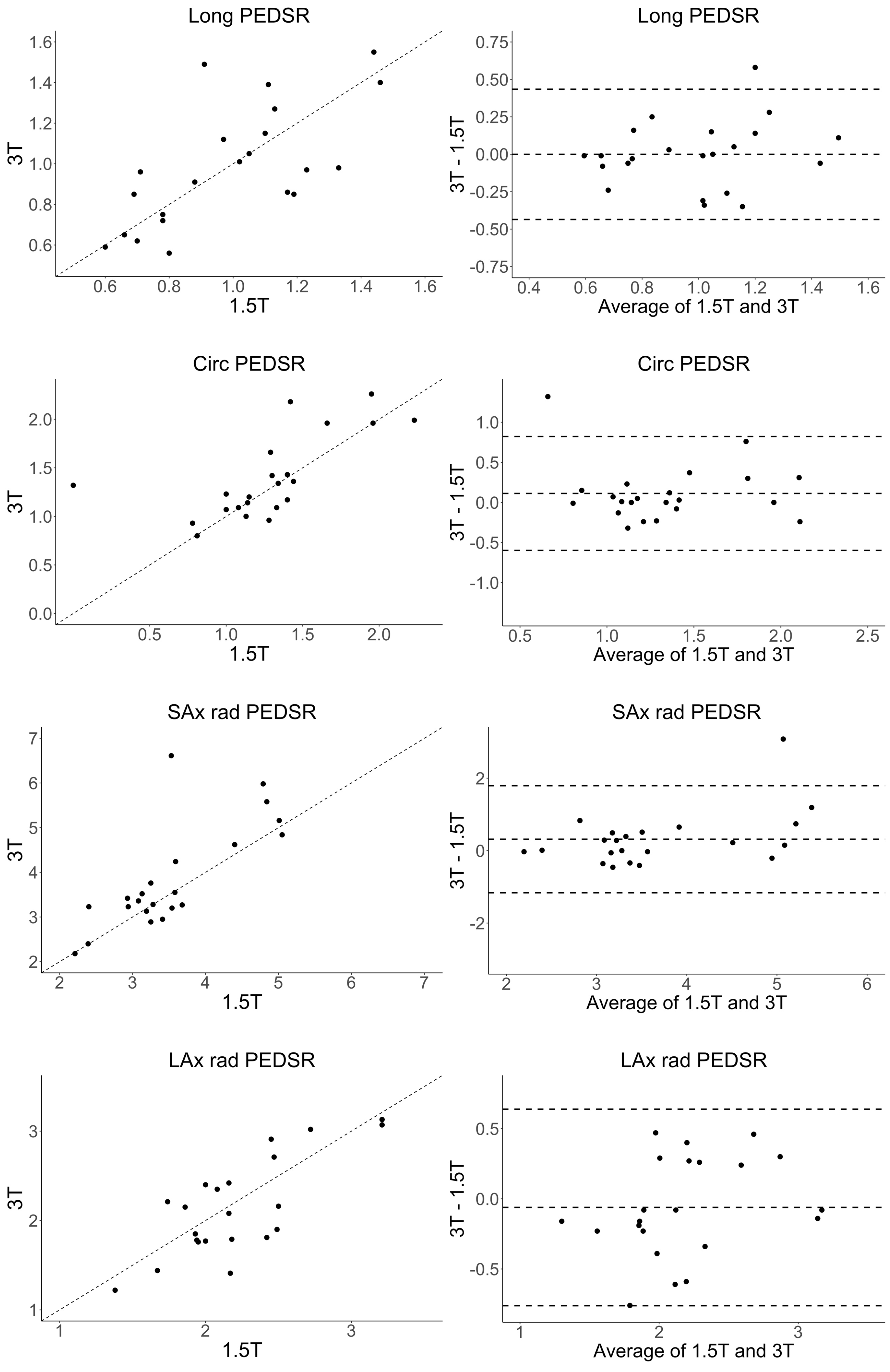

Supplementary figure 7: Inter-field strength agreement of LV PEDSR using QStrain

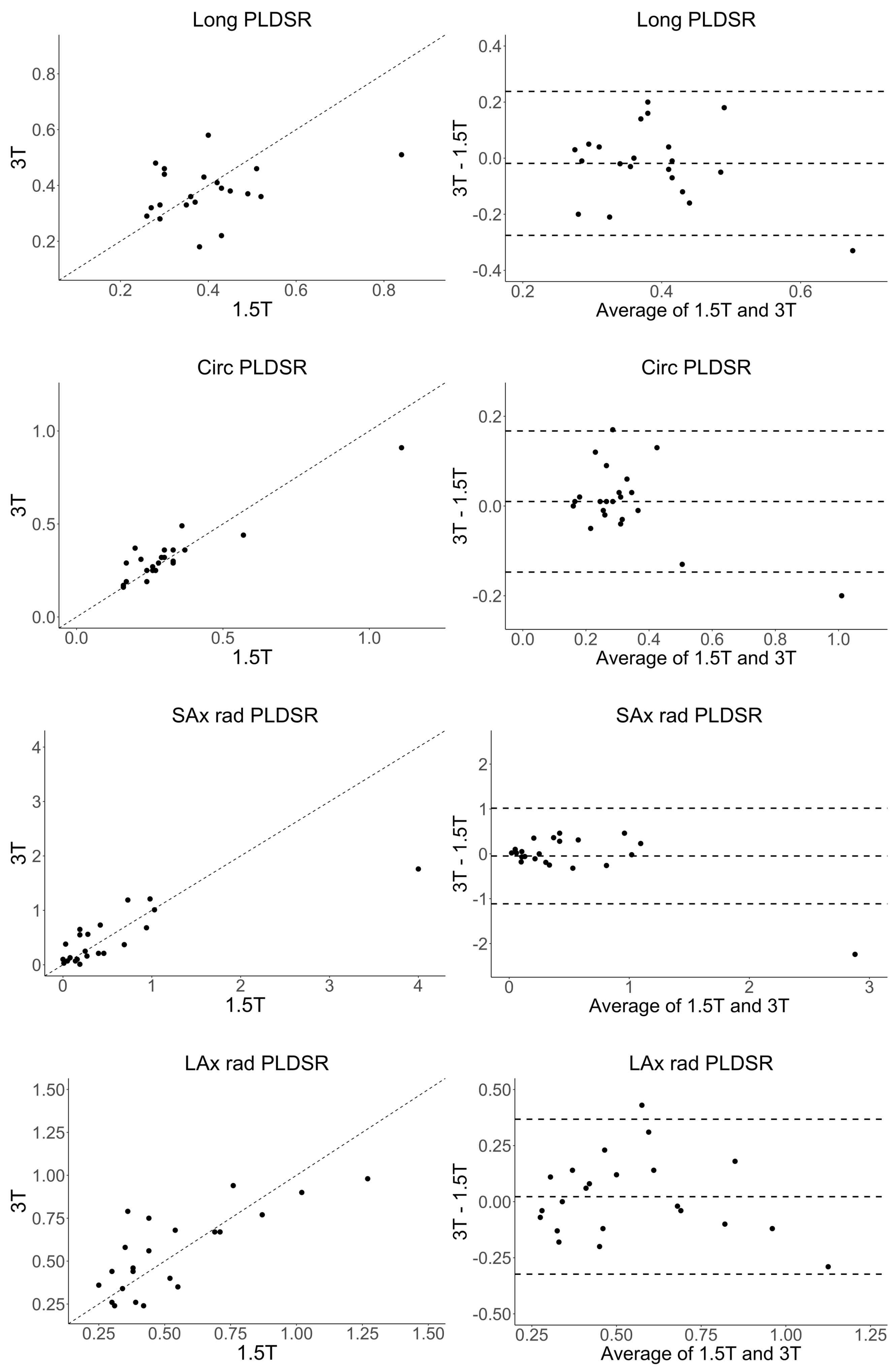

Supplementary figure 8: Inter-field strength agreement of LV PLDSR using QStrain
