# Supplementary material for: The Interfield Strength Agreement of Left Ventricular Strain Measurements at 1.5 T and 3 T Using Cardiac MRI Feature Tracking
Source: J Magn Reson Imaging. 2022 Jun 29;57(4):1250–61. doi: 10.1002/jmri.28328 (PMC10947203; doi:10.1002/jmri.28328)
Supplement: Supplementary file 4 — Additional file 4 Title and description of data: Supplementary Table 2: Sensitivity analysis for the inter‐field strength agreement of left ventricular strain and strain rate measurements using cvi42 Tissue Tracking at 1.5 T and 3 T using excellent and very good image quality scans (n = 18 for long axis images and n = 18 for short axis images) [file JMRI-57-1250-s007.pdf]

**<u>Supplementary Table 2</u>**: Sensitivity analysis for the inter-field strength agreement of left ventricular strain and strain rate measurements using cvi42 Tissue Tracking at 1.5T and 3T using excellent and very good image quality scans (n = 18 for long axis images and n = 18 for short axis images)

| | 1.5T | 3T | P value | r | ICC |
|---|---|---|---|---|---|
| GLS (%) | 17.77 (16.83, 18.57) | 16.42 (15.35, 18.87) | 0.64 | 0.48 | 0.49 |
| GCS (%) | 18.70 (17.43, 20.49) | 19.27 (17.61, 20.86) | 0.18 | 0.84 | 0.81 |
| Short axis GRS (%) | 31.78 (28.17, 36.62) | 33.45 (29.62, 37.88) | 0.14 | 0.84 | 0.73 |
| Long axis GRS (%) | 31.94 (28.11, 36.01) | 27.96 (26.03, 34.41) | 0.44 | 0.47 | 0.44 |
| Longitudinal PSSR (1/s) | 0.88 (0.77, 0.93) | 0.85 (0.74, 0.98) | 0.87 | 0.39 | 0.41 |
| Circumferential PSSR (1/s) | 0.98 (0.91, 1.04) | 0.97 (0.93, 1.08) | 0.35 | 0.80 | 0.81 |
| Short axis radial PSSR (1/s) | 1.63 (1.45, 2.02) | 1.54 (1.45, 1.91) | 0.77 | 0.79 | 0.69 |
| Long axis radial PSSR (1/s) | 1.66 (1.30, 1.74) | 1.65 (1.34, 1.83) | 0.42 | 0.40 | 0.26 |
| Longitudinal PEDSR (1/s) | 0.93 (0.77, 1.06) | 0.85 (0.68, 1.04) | 0.61 | 0.38 | 0.48 |
| Circumferential PEDSR (1/s) | 1.22 (1.02, 1.32) | 1.21 (0.93, 1.43) | 0.17 | 0.89 | 0.84 |
| Short axis radial PEDSR (1/s) | 2.20 (1.94, 2.85) | 2.27 (1.68, 2.71) | 0.70 | 0.77 | 0.83 |
| Long axis radial PEDSR (1/s) | 2.16 (1.57, 2.51) | 1.85 (1.48, 2.20) | 0.42 | 0.48 | 0.48 |
| Longitudinal PLDSR (1/s) | 0.47 (0.40, 0.62) | 0.46 (0.36, 0.59) | 0.18 | 0.69 | 0.63 |
| Circumferential PLDSR (1/s) | 0.38 (0.32, 0.49) | 0.43 (0.34, 0.51) | 0.16 | 0.82 | 0.90 |
| Short axis radial PLDSR (1/s) | 0.51 (0.43, 0.69) | 0.51 (0.40, 0.67) | 0.30 | 0.82 | 0.91 |

| | 1.5T | 3T | P value | r | ICC |
|---|---|---|---|---|---|
| Long axis radial PLDSR (1/s) | 0.51 (0.45, 0.75) | 0.60 (0.45, 0.67) | 0.49 | 0.76 | 0.70 |
| Peak torsion (deg/cm) | 1.43 (1.16, 1.75) | 1.28 (1.08, 1.64) | 0.44 | 0.31 | 0.32 |

Median (IQR)

GCS, global circumferential strain; GLS, global longitudinal strain; GRS, global radial strain; ICC, intraclass correlation co-efficient; PEDSR, peak early diastolic strain rate; PLDSR, peak late diastolic strain rate; PSSR, peak systolic strain rate; r, Spearman's correlation co-efficient
