# Supplementary material for: The Interfield Strength Agreement of Left Ventricular Strain Measurements at 1.5 T and 3 T Using Cardiac MRI Feature Tracking
Source: J Magn Reson Imaging. 2022 Jun 29;57(4):1250–61. doi: 10.1002/jmri.28328 (PMC10947203; doi:10.1002/jmri.28328)
Supplement: Supplementary file 5 — Additional file 5 Title and description of data: Supplementary Table 3: Intra‐observer variability of strain and strain rate measurements using cvi42 Tissue Tracking at 1.5 T and 3 T Supplementary Table 4: Inter‐observer variability of strain and strain rate measurements using cvi42 Tissue Tracking at 1.5 T and 3 T [file JMRI-57-1250-s002.pdf]

Supplementary Table 3: Intra-observer variability of strain and strain rate measurements using cvi42 Tissue Tracking at 1.5T and 3T

	1.5T					3T				
	Bias	Limits of agreement	ICC	r	p value	Bias	Limits of agreement	ICC	r	p value
GLS (%)	-0.43	-2.16, 1.29	0.92	0.98	0.29	-0.20	-2.04, 1.64	0.91	0.73	0.38
GCS (%)	-0.21	-1.36, 0.94	0.97	0.81	0.24	-0.06	-1.11, 1.00	0.98	0.98	0.81
Short axis GRS (%)	-0.63	-3.22, 1.96	0.98	0.84	0.11	-0.18	-2.73, 2.37	0.98	0.96	0.77
Long axis GRS (%)	-1.52	-6.71, 3.67	0.91	0.94	0.11	-1.74	-5.38, 1.90	0.90	0.96	0.02
Longitudinal PSSR (1/s)	-0.06	0.08, -0.19	0.77	0.86	0.04	0.002	0.25, -0.25	0.77	0.83	0.92
Circumferential PSSR (1/s)	-0.05	0.13, -0.22	0.78	0.21	0.13	0.0006	0.13, -0.13	0.84	0.48	>0.99
Short axis radial PSSR 1/s)	-0.03	0.21, -0.28	0.96	0.82	0.56	0.03	0.25, -0.19	0.96	0.71	0.38
Long axis radial PSSR (1/s)	-0.09	0.15, -0.33	0.90	0.89	0.084	-0.14	0.44, -0.72	0.61	0.65	0.13
Longitudinal PEDSR (1/s)	-0.02	0.26, -0.31	0.88	0.89	0.77	-0.04	0.09, -0.17	0.94	0.92	0.084
Circumferential PEDSR (1/s)	-0.03	0.13, -0.18	0.94	0.89	0.51	-0.002	0.12, -0.13	0.98	0.98	>0.99
Short axis radial PEDSR 1/s)	-0.09	0.25, -0.43	0.95	0.80	0.23	0.01	0.33, -0.31	0.98	0.95	0.70
Long axis radial PEDSR (1/s)	-0.12	0.51, -0.75	0.90	0.97	0.23	-0.16	0.22, -0.54	0.90	0.92	0.03
Longitudinal PLDSR (1/s)	-0.04	0.11, -0.19	0.75	0.80	0.084	0.04	0.17, -0.09	0.87	0.78	0.084
Circumferential PLDSR (1/s)	-0.001	0.08, -0.08	0.92	0.84	>0.99	0.006	0.06, -0.05	0.97	0.90	0.49
Short axis radial PLDSR 1/s)	0.002	0.07, -0.06	0.96	0.88	0.92	0.007	0.07, -0.06	0.97	0.92	0.64

Long axis radial PLDSR (1/s)	-0.05	0.14, -0.23	0.77	0.82	0.15	0.04	0.18, -0.10	0.91	0.84	0.13
Torsion (deg/cm)	-0.16	0.77, -1.08	0.62	0.48	0.11	-0.003	1.06, -1.06	0.57	0.41	0.92

GCS, global circumferential strain; GLS, global longitudinal strain; GRS, global radial strain; ICC, intraclass correlation; PEDSR, peak early diastolic strain rate; PLDSR, peak late diastolic strain rate; PSSR, peak systolic strain rate; LV, left ventricular; r, Spearman's correlation co-efficient

Supplementary Table 4: Inter-observer variability of strain and strain rate measurements using cvi42 Tissue Tracking at 1.5T and 3T

	1.5T					3T				
	Bias	Limits of agreement	ICC	r	p value	Bias	Limits of agreement	ICC	r	p value
GLS (%)	0.16	2.60, -2.28	0.84	0.73	0.49	0.49	3.13, -2.16	0.61	0.60	0.38
GCS (%)	0.74	1.48, -0.008	0.94	0.99	0.004	0.53	1.52, -0.47	0.95	1.00	0.02
Short axis GRS (%)	1.97	4.24, -0.30	0.94	0.99	0.004	1.74	4.73, -1.26	0.95	0.98	0.004
Long axis GRS (%)	0.86	8.25, -6.53	0.81	0.79	0.38	1.01	6.63, -4.62	0.64	0.72	0.23
Longitudinal PSSR (1/s)	0.09	0.38, -0.21	0.79	0.82	0.11	0.13	0.38, -0.14	0.70	0.73	0.02
Circumferential PSSR (1/s)	0.05	0.17, -0.07	0.87	0.89	0.04	0.03	0.18, -0.12	0.93	0.92	0.19
Short axis radial PSSR 1/s)	0.12	0.28, -0.04	0.92	0.96	0.004	0.06	0.23, -0.10	0.98	0.94	0.066
Long axis radial PSSR (1/s)	0.11	0.74, -0.52	0.67	0.69	0.32	0.15	0.60, -0.30	0.73	0.79	0.064
Longitudinal PEDSR (1/s)	-0.005	0.17, -0.18	0.93	0.94	>0.99	0.06	0.32, -0.19	0.75	0.89	0.16
Circumferential PEDSR (1/s)	0.06	0.21, -0.09	0.94	0.94	0.02	0.02	0.19, -0.14	0.95	0.90	0.49
Short axis radial PEDSR 1/s)	0.22	0.55, -0.11	0.91	0.95	0.006	0.23	0.65, -0.18	0.87	0.96	0.01
Long axis radial PEDSR (1/s)	0.09	0.75, -0.57	0.85	0.80	0.49	0.13	0.63, -0.38	0.77	0.79	0.064
Longitudinal PLDSR (1/s)	-0.01	0.16, -0.18	0.88	0.77	0.62	0.009	0.18, -0.17	0.73	0.59	0.62
Circumferential PLDSR (1/s)	0.006	0.06, -0.05	0.99	0.94	0.44	0.04	0.13, -0.06	0.94	0.95	0.084
Short axis radial PLDSR 1/s)	0.008	0.07, -0.06	0.99	0.94	0.49	0.04	0.15, -0.06	0.95	0.95	0.04

Long axis radial PLDSR (1/s)	0.0004	0.2, -0.21	0.9	0.78	>0.99	0.011	0.19, -0.16	0.82	0.90	0.49
Torsion (deg/cm)	0.12	1.49, -1.24	0.42	0.29	>0.99	0.26	1.91, -1.40	0.43	0.49	0.23

GCS, global circumferential strain; GLS, global longitudinal strain; GRS, global radial strain; ICC, intraclass correlation; PEDSR, peak early diastolic strain rate; PLDSR, peak late diastolic strain rate; PSSR, peak systolic strain rate; LV, left ventricular; r, Spearman's correlation co-efficient
