# Supplementary material for: The Interfield Strength Agreement of Left Ventricular Strain Measurements at 1.5 T and 3 T Using Cardiac MRI Feature Tracking
Source: J Magn Reson Imaging. 2022 Jun 29;57(4):1250–61. doi: 10.1002/jmri.28328 (PMC10947203; doi:10.1002/jmri.28328)
Supplement: Supplementary file 7 — Additional file 7 Title and description of data: Supplementary figure 14: Inter‐observer variability of LV global strain GCS, global circumferential strain; GLS, global longitudinal strain; GRS, global radial strain; LAx, long axis; LV, left ventricular; SAx, short axis Supplementary figure 15: Inter‐observer variability of LV PSSR LAx, long axis; LV, left ventricular; PSSR, peak systolic strain rate; SAx, short axis Supplementary figure 16: Inter‐observer variability of LV PEDSR LAx, long axis; LV, left ventricular; PEDSR, peak early diastolic strain rate; SAx, short axis Supplementary figure 17: Inter‐observer variability of LV PLDSR LAx, long axis; LV, left ventricular; PLDSR, peak late diastolic strain rate; SAx, short axis Supplementary figure 18: Inter‐observer variability of peak torsion [file JMRI-57-1250-s006.pdf]

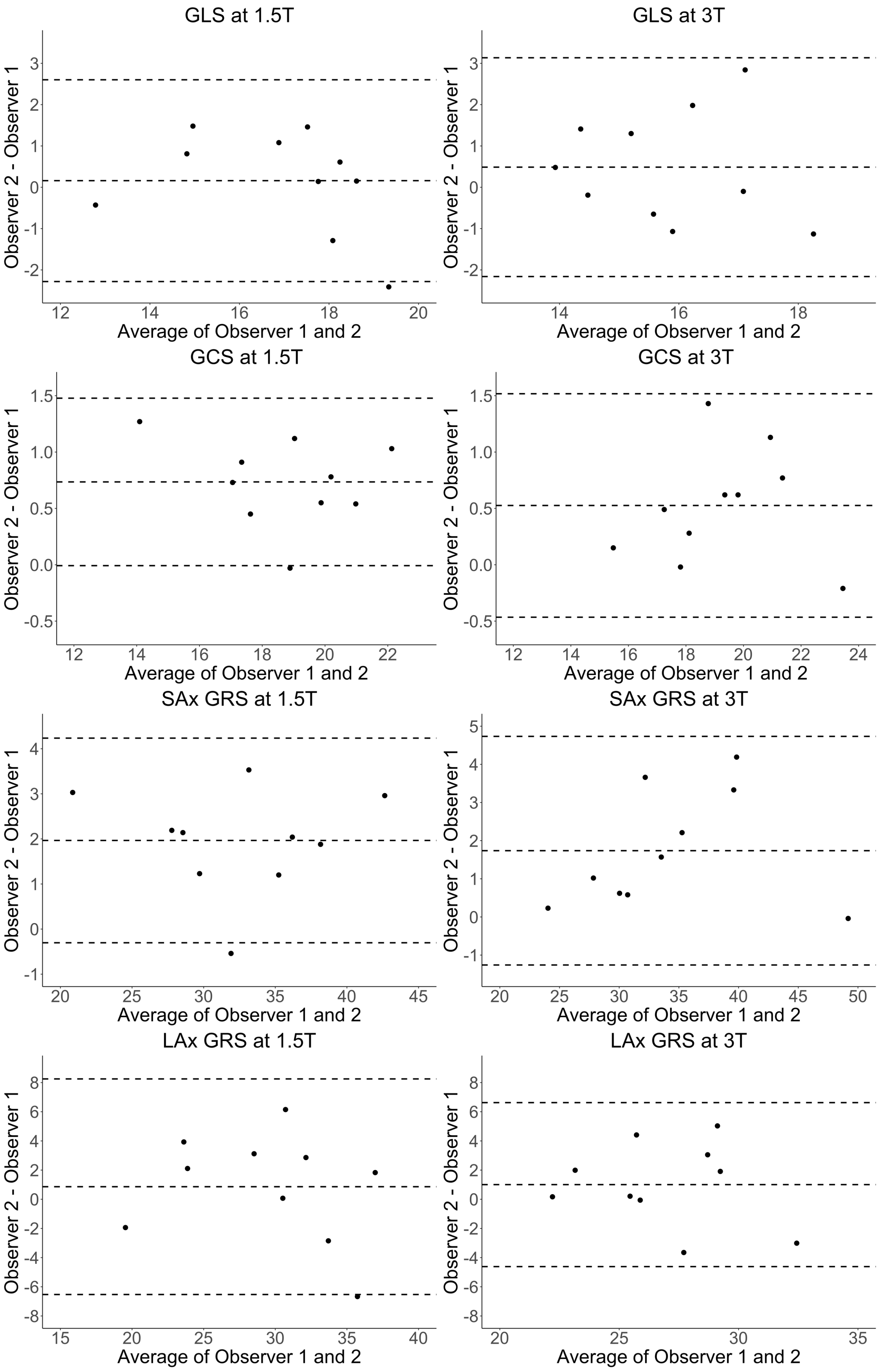

Supplementary figure 14: Inter-observer variability of LV global strain

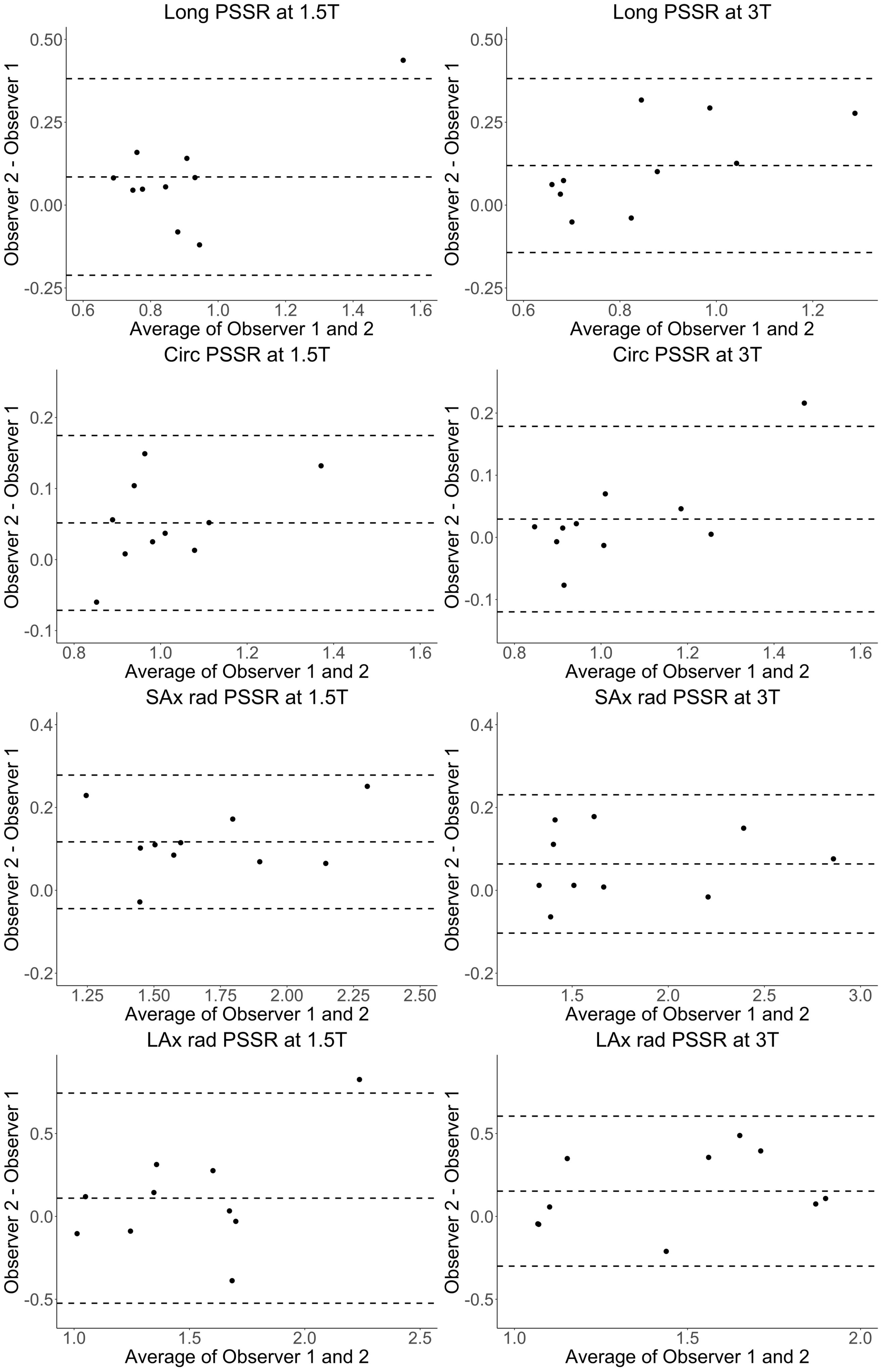

Supplementary figure 15: Inter-observer variability of LV global PSSR

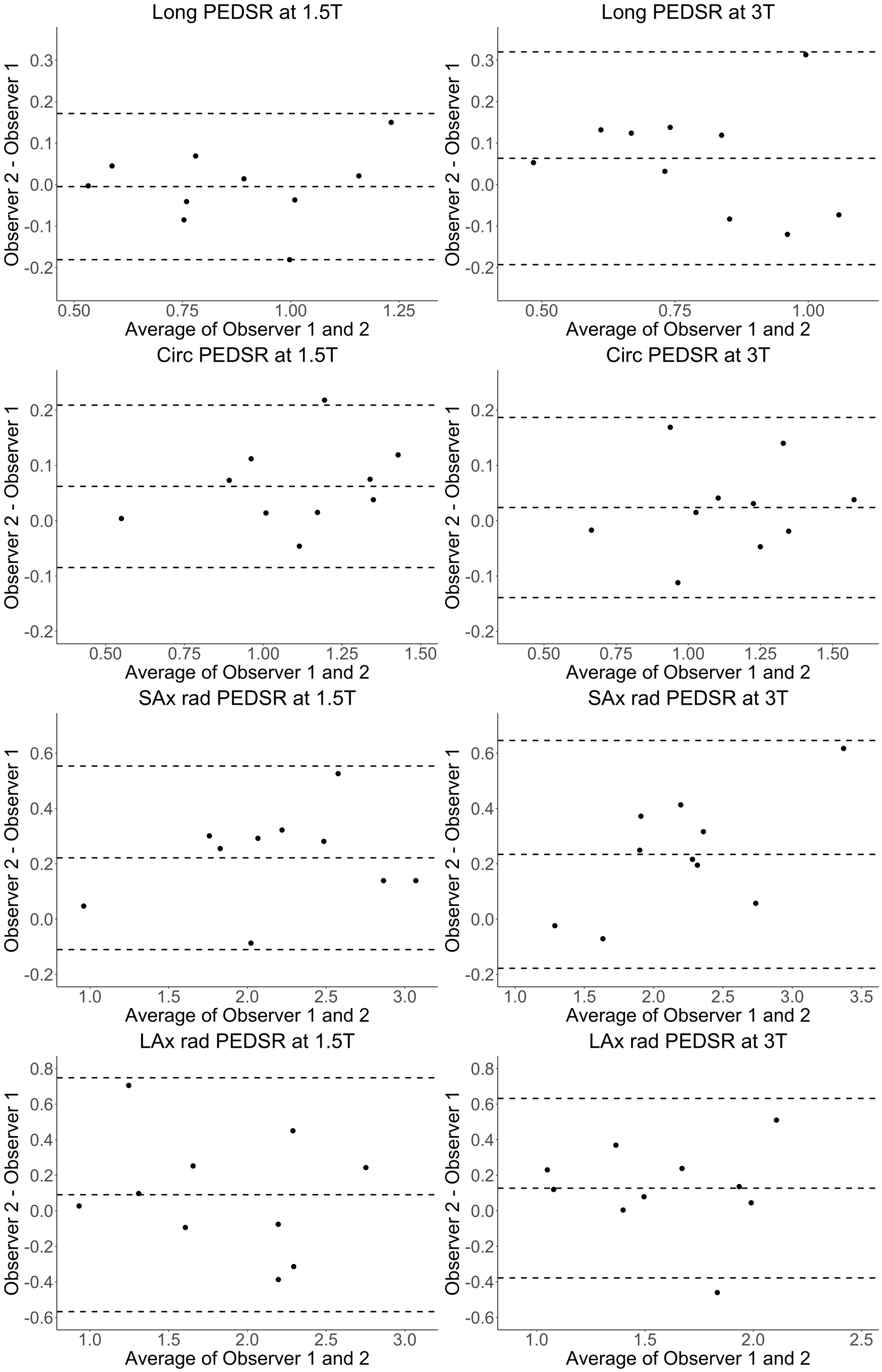

Supplementary figure 16: Inter-observer variability of LV global PEDSR

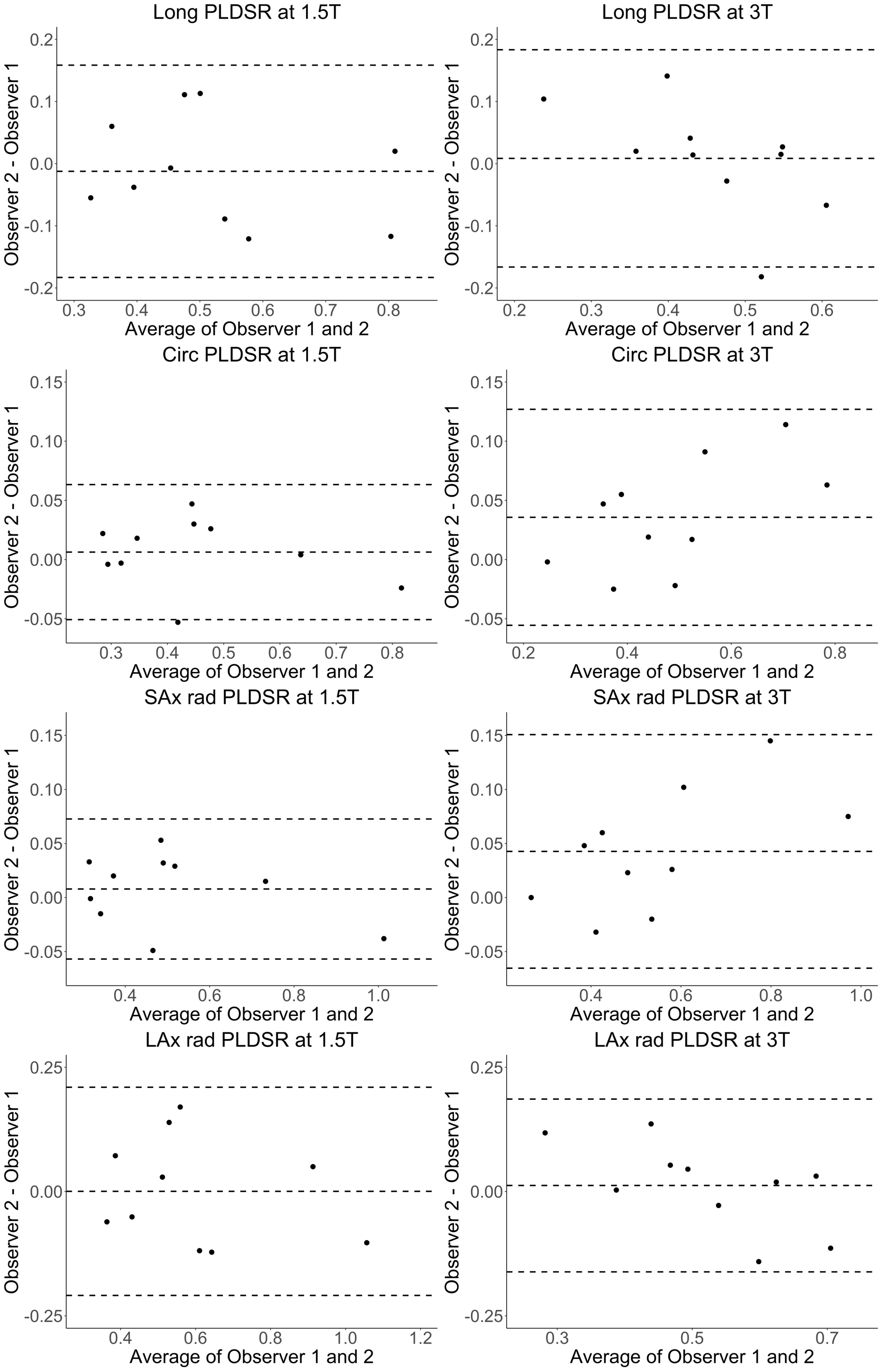

Supplementary figure 17: Inter-observer variability of LV global PLDSR

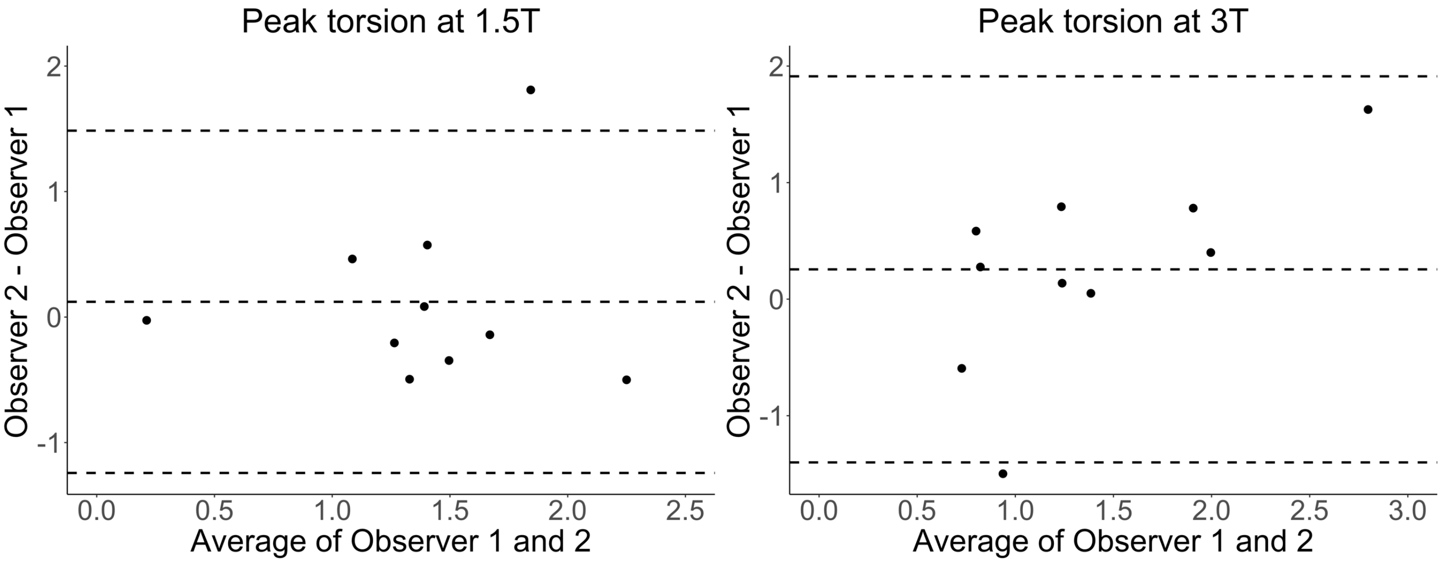

Supplementary figure 18: Inter-observer variability of peak torsion
